# The effect of background noise and its removal on the analysis of single-cell expression data

**DOI:** 10.1186/s13059-023-02978-x

**Published:** 2023-06-19

**Authors:** Philipp Janssen, Zane Kliesmete, Beate Vieth, Xian Adiconis, Sean Simmons, Jamie Marshall, Cristin McCabe, Holger Heyn, Joshua Z. Levin, Wolfgang Enard, Ines Hellmann

**Affiliations:** 1 Anthropology and Human Genomics, Faculty of Biology, Ludwig-Maximilians University, Munich, Germany; 2 Klarman Cell Observatory, Broad Institute of Harvard and MIT, Cambridge, USA; 3 Stanley Center for Psychiatric Research, Broad Institute of Harvard and MIT, Cambridge, USA; 4 Broad Institute of Harvard and MIT, Cambridge, USA; 5 CNAG-CRG, Centre for Genomic Regulation, Barcelona Institute of Science and Technology, Barcelona, Spain

**Keywords:** Single-cell RNA-sequencing, Background noise, Ambient RNA, Barcode swapping, Correction method comparison, (Gold) standard scRNA-seq data set

## Abstract

**Background:**

In droplet-based single-cell and single-nucleus RNA-seq experiments, not all reads associated with one cell barcode originate from the encapsulated cell. Such background noise is attributed to spillage from cell-free ambient RNA or barcode swapping events.

**Results:**

Here, we characterize this background noise exemplified by three scRNA-seq and two snRNA-seq replicates of mouse kidneys. For each experiment, cells from two mouse subspecies are pooled, allowing to identify cross-genotype contaminating molecules and thus profile background noise. Background noise is highly variable across replicates and cells, making up on average 3–35% of the total counts (UMIs) per cell and we find that noise levels are directly proportional to the specificity and detectability of marker genes. In search of the source of background noise, we find multiple lines of evidence that the majority of background molecules originates from ambient RNA. Finally, we use our genotype-based estimates to evaluate the performance of three methods (CellBender, DecontX, SoupX) that are designed to quantify and remove background noise. We find that CellBender provides the most precise estimates of background noise levels and also yields the highest improvement for marker gene detection. By contrast, clustering and classification of cells are fairly robust towards background noise and only small improvements can be achieved by background removal that may come at the cost of distortions in fine structure.

**Conclusions:**

Our findings help to better understand the extent, sources and impact of background noise in single-cell experiments and provide guidance on how to deal with it.

**Supplementary Information:**

The online version contains supplementary material available at 10.1186/s13059-023-02978-x.

## Background

Single cell and single nucleus RNA-seq (scRNA-seq, snRNA-seq) are in the process of revolutionizing medical and biological research. The typically sparse coverage per cell and gene is compensated by the capability of analyzing thousands of cells in one experiment. In droplet-based protocols such as 10x Chromium, this is achieved by encapsulating single cells in droplets together with beads that carry oligonucleotides. These usually consist of a oligo(dT) sequence which is used for priming reverse transcription, a bead-specific barcode that tags all transcripts encapsulated within the droplet as well as unique molecular identifiers (UMIs) that enable the removal of amplification noise [1–3]. As proof of principle that each droplet encapsulates only one cell, it is common to use mixtures of cells from human and mouse [3]. Thus doublets, i.e., droplets containing two cells, can be readily identified as they have an approximately even mixture of mouse and human transcripts. However, barcodes for which the clear majority of reads is either mouse or human, still contain a small fraction of reads from the other species [3–5]. Furthermore, presumably empty droplets also yield sequence reads [4].

One potential source of such contaminating reads or background noise is cell-free “ambient” RNA that leaked from broken cells into the suspension. The other potential source are chimeric cDNA molecules that can arise during library preparation due to so-called ’barcode swapping’. The pooling of barcode tagged cDNA after reverse transcription but before PCR amplification, is a decisive step to achieve high throughput. However, if amplification of tagged cDNA molecules occurs from unremoved oligonucleotides from other beads or from incompletely extended PCR products (originally called template jumping [6]), this generates a chimeric molecule with a “swapped” barcode and UMI [7, 8]. When sequencing this molecule, the cDNA is assigned to the wrong barcode and hence “contaminates” the expression profile of a cell. However, unless the swapping occurs between two different genes, the barcode and UMI will still be counted correctly. Another type of barcode swapping can occur during PCR amplification on a patterned Illumina flowcell before sequencing [9] with the same effects, although double indexing of Illumina libraries has reduced this problem substantially. This said, here we focus on barcode swapping that occurs during library preparation.

Irrespective of the source of background noise, its presence can interfere with analyses. For starters, background noise reduces the separability of cell type clusters as well as the power to pinpoint important (marker) genes via differential expression analysis. Moreover, reads from cell type-specific marker genes spill over to cells of other types, thus yielding novel marker combinations and hence implying the presence of novel cell types [8, 10]. Besides, background noise can also confound differential expression analysis between samples, e.g., when looking for expression changes within a cell type between two conditions. Varying amounts of background noise or differences in the cell type composition between conditions can result in dissimilar background profiles, which might generate false positives when identifying differentially expressed genes. To alleviate such problems during downstream analysis, algorithms to estimate and correct for the amounts of background noise have been developed.

SoupX estimates the contamination fraction per cell using marker genes and then deconvolutes the expression profiles using empty droplets as an estimate of the background noise profile [11]. In contrast, DecontX defaults to model the fraction of background noise in a cell by fitting a mixture distribution based on the clusters of good cells [8], but also allows the user to provide a custom background profile, e.g., from empty droplets. CellBender requires the expression profiles measured in empty droplets to estimate the mean and variance of the background noise profile originating from ambient RNA. In addition, CellBender explicitly models the barcode swapping contribution using mixture profiles of the ’good’ cells [4].

In order to evaluate method performance, one dataset of an even mix between one mouse and one human cell line [3] is commonly used to get an experimentally determined lower bound of background noise levels that is identified as counts covering genes from the other species [4, 8, 11, 12]. Since this dataset is lacking in cell type diversity, it is common to additionally evaluate performance based on other datasets that have a complex cell type mixture and where most cell types have well known profiles with exclusive marker genes. In such studies the performance test is whether the model removes the expression of the exclusive marker genes from the other cell types. In both cases, the feature space of the contamination does not overlap with the endogenous cell feature space. Mouse and human are too diverged, so that mouse reads only map to mouse genes and human reads only to human genes. Similarly, when using marker genes it is assumed that they are exclusively expressed in only one cell type, hence the features that are used for background inference are again not overlapping. However, in reality background noise will mostly induce shifts in expression levels that cannot be described in a binary on or off sense and it remains unclear how background correction will affect those profiles.

Here, we use a mouse kidney dataset representing a complex cell type mixture from three mouse strains of two subspecies, *Mus musculus domesticus* and *M. m. castaneus*. From both subspecies, inbred strains were used and thus we can distinguish exogenous and endogenous counts for the same features using known homozygous SNPs [13]. Hence, this dataset serves as a much more realistic experimental standard, providing a ground truth in a complex setting with multiple cell types which allows to analyze the variability, the source and the impact of background noise on single cell analysis. Moreover, this dataset enables us to better benchmark existing background removal methods.

## Results

### Mouse kidney single cell and single nucleus RNA-seq data

**Fig. 1:**
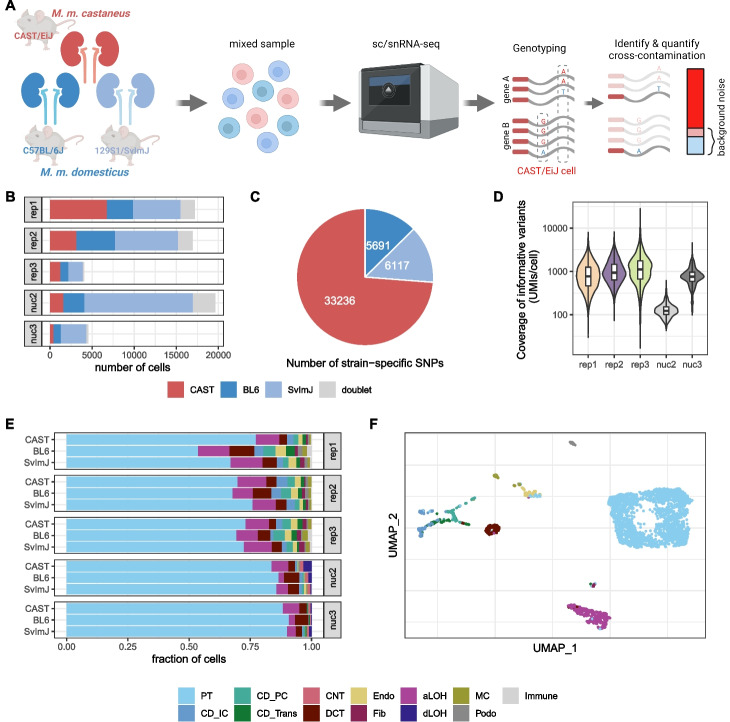
Generation of mouse strain mixture datasets to quantify background noise. **A** Experimental design (created with BioRender.com). **B** Strain composition in 5 different replicates, subjected to scRNA-seq (rep1-3) or snRNA-seq (nuc2, nuc3). The replicates rep2 and nuc2 and rep3 and nuc3 were generated from the same samples each. CAST: CAST/EiJ strain; BL6: C57BL/6J strain; SvImJ: 129S1/SvImJ. **C** Number of homozygous SNPs with a coverage of more than 100 UMIs that distinguish one strain from the other two. **D** Per cell coverage in *M. m. castaneus* cells of informative variants that distinguish *M. m. castaneus* and *M. m. domesticus.* **E** Cell type composition per replicate and strain; labels were obtained by reference-based classification using mouse kidney data from Denisenko et al. [14] as reference. **F** UMAP visualization of *M. m. castaneus* cells in single-cell replicate 2, colored by assigned cell type. PT, proximal tubule; CD_IC, intercalated cells of collecting duct; CD_PC, principal cells of collecting duct; CD_Trans, transitional cells of collecting duct; CNT, connecting tubule; DCT, distal convoluted tubule; Endo, endothelial; Fib, fibroblasts; aLOH, ascending loop of Henle; dLOH, descending loop of Henle; MC, mesangial cells; Podo, podocytes

We obtained three replicates for single cell RNA-seq (rep1-3) data and two replicates for single nucleus RNA-seq (snRNA-seq, nuc2 and nuc3) data from the same samples that were used in scRNA-seq replicates 2 and 3, respectively. Each replicate consists of one channel of 10× [3] in which cells from dissociated kidneys of three mice each were pooled: one *M. m. castaneus* from the strain CAST/EiJ (CAST) and two *M. m. domesticus*, one from the strain C57BL/6J (BL6) and one from the strain 129S1/SvImJ (SvImJ) (Fig. 1A). Based on known homozygous SNPs that distinguish subspecies and strains, we assigned cells to mice (Fig. 1B). In total, we identified $>40,000$ informative SNPs of which the majority (32,000) separates the subspecies and $\sim 10,000$ SNPs distinguish the two *M. m. domesticus* strains (Fig. 1C). On average, each cell had sufficient coverage for $\sim 1,000$ informative SNPs ($\sim 20\%$ of total UMIs per cell) to provide us with unambiguous genotype calls for those sites. The coverage for the nuc2 data was much lower with only $\sim 100$ SNPs (Fig. 1D).

Overall, each experiment yielded 5000–20,000 good cells with 9–43% *M. m. castaneus* (Fig. 1B). Thus, the majority of background noise in any *M. m. castaneus* cell is expected to be from *M. m. domesticus* (Additional file 1: Fig. S1B) and therefore we expect that genotype-based estimates of cell-wise amounts of background noise for *M. m. castaneus* to be fairly accurate (Additional file 1: Fig. S2). Hence from here on out we focus on *M. m. castaneus* cells for the analysis of the origins of background noise and also as the ground truth for benchmarking background removal methods.

This dataset has two advantages over the commonly used mouse-human mix [3]. Firstly, the kidney data have a high cell type diversity. Using the data from Denisenko et al. [14] as reference dataset for kidney cell types, we could identify 13 cell types. Encouragingly, the cell type composition is very similar across mouse strains as well as replicates with proximal tubule cells constituting 66–89% of the cells (Fig. 1E, F; Additional file 1: Fig. S3). Secondly, due to the higher similarity of the mouse subspecies, we can identify contaminating reads for the same features. $\sim 7,000$ genes carry at least one informative SNP about the subspecies. Because so many genes have informative SNPs, the fraction of UMIs that cover an informative SNP is a little higher for PTs, the most frequent cell type, but very comparable across all other cell types, allowing us to quantify contaminating reads (Additional file 1: Fig. S1A).

### Background noise fractions differ between replicates and cells

**Fig. 2:**
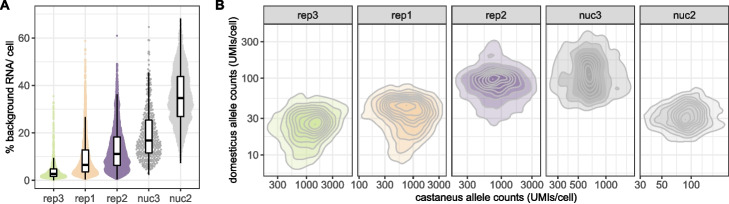
The level of background noise is variable across replicates and single cells. **A** Estimated fraction of background noise per cell. The replicates on the *x*-axis are ordered by ascending median background noise fraction. **B** In *M. m. castaneus* cells both endogenous *M. m. castaneus* specific alleles (*x*-axis) and *M. m. domesticus* specific alleles (*y*-axis) have coverage in each cell. The detection of *M. m. domesticus* specific alleles can be seen as background noise originating from cells of a different mouse

Around 5–20% of the UMI counts are from molecules that contain a SNP that is informative about the subspecies of origin. We quantify in each *M. m. castaneus* cell how often an endogenous *M. m. castaneus* allele or a foreign *M. m. domesticus* allele was covered. Assuming that the count fractions covering the SNPs are representative of the whole cell, we detect a median of 2–27% counts from the foreign genotype over all cells per experiment (Additional file 1: Fig. S1C). This observed cross-genotype contamination fraction represents a lower bound of the overall amounts of background noise. As suggested in Heaton et al. [15], we then integrate over the foreign allele fractions of all informative SNPs to obtain a maximum likelihood estimate of the background noise fraction ($\rho _{cell}$) of each cell that extrapolates to also include contamination from the same genotype (see the “Methods” section, Additional file 1: Fig. S2). Based on these estimates, we find that background noise levels vary considerably between replicates and do not appear to depend on the overall success of the experiment measured as the cell yield per lane (Fig. 2). For example in scRNA-seq rep3 (3900 cells), we detected overall the fewest good cells, but most of those cells had less than 3% background noise, while the much more successful rep2 (15,000 cells) we estimated the median background noise level at around 11% (Fig. 2A). This said, the snRNA-seq data generated from frozen tissue have much higher background levels than the corresponding scRNA-seq replicates — 35% in nuc2 vs. 11% rep2 and 17% in nuc3 vs. 3% in rep3. How we define good cells based on the UMI counts has little impact on this variability. We still find by far the highest background levels in nuc2 and the lowest in rep3 (Additional file 1: Fig. S4). This high variability is not very surprising. This being a real life experiment and experimental conditions were improved for nuc3 based on the experience with nuc2 (see the “Methods” section). The number of contaminating RNA-molecules (UMIs) depends only weakly on the total UMI counts covering informative variants as a proxy for sequencing depth of the cell (Fig. 2B, Additional file 1: Table S1). Such a weak correlation could be explained by variation in the capture efficiency in each droplet. An alternative, but not mutually exclusive explanation of such a correlation could be that the source of some contaminating molecules is barcode swapping that can occur during library amplification.

However, by and large the absolute amount of background noise is approximately constant across cells and thus the contamination fraction mainly depends on the amount of endogenous RNA: the larger the cell, the smaller the fraction of background noise, pointing towards ambient RNA as the major source of the detected background (Fig. 2B).

### Contamination profiles show a high similarity to ambient RNA profiles

**Fig. 3:**
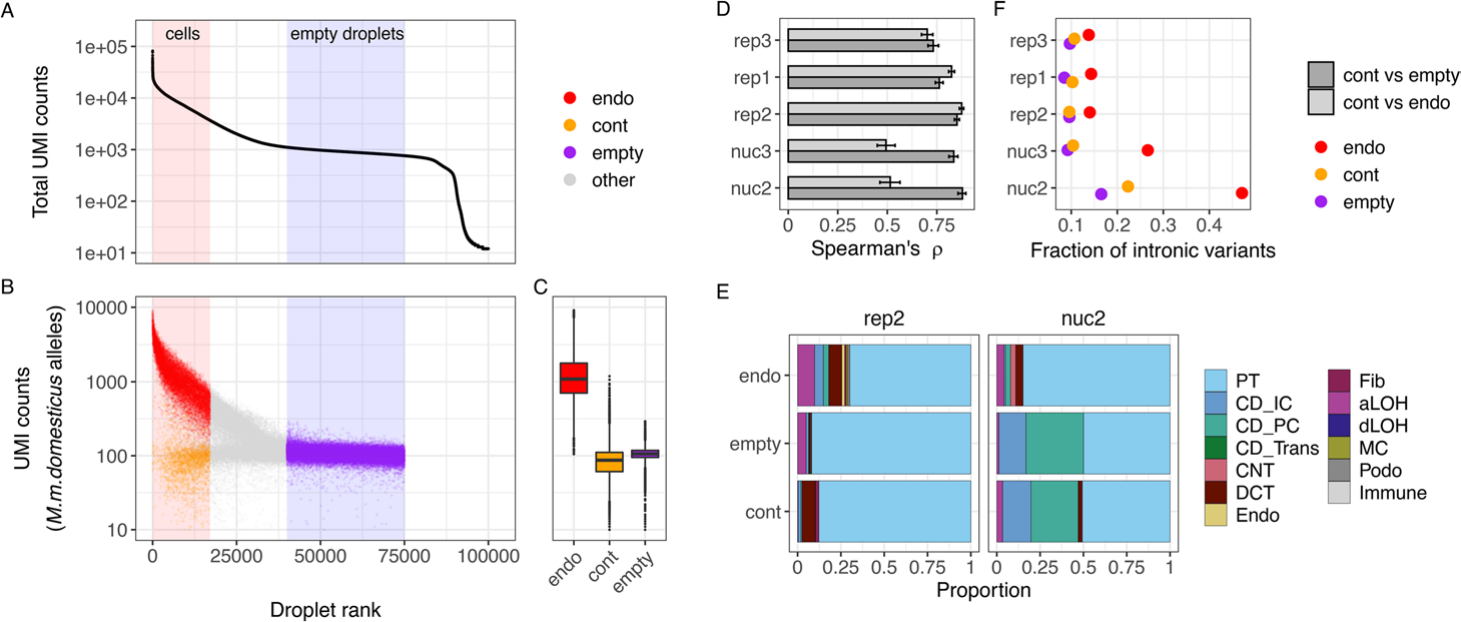
Characterization of ambient RNA in cells and empty droplets. **A** Ordering droplet barcodes by their total UMI count to distinguish cell-containing droplets with high UMI counts from empty droplets that only contain cell-free ambient RNA and are identifiable as a plateau in the UMI curve, shown here for replicate 2. **B** UMI counts of reads covering *M. m. domesticus* specific alleles were used to construct three profiles depending on whether they were associated with *M. m. domesticus* cell barcodes (endogenous counts, endo), *M. m. castaneus* cell barcodes (contaminating counts, cont) or empty droplet barcodes (empty). Counts from droplets that are not clearly assignable as cell-containing or empty were excluded from further analysis (other). **C** UMI counts per cell for each of the three profiles. **D** Spearman rank correlation between pseudobulk profiles. Error bars indicate 95% confidence intervals obtained by bootstrapping over genes. **E** Deconvolution of cell type contributions to each pseudobulk profile, exemplified by replicates rep2 and nuc2. The stacked barplots depict the estimated fraction of each cell type in the profile as inferred by SCDC using the annotated single cell data of each replicate as reference. PT, proximal tubule; CD_IC, intercalated cells of collecting duct; CD_PC, principal cells of collecting duct; CD_Trans, transitional cells of collecting duct; CNT, connecting tubule; DCT, distal convoluted tubule; Endo, endothelial; Fib, fibroblasts; aLOH, ascending loop of Henle; dLOH, descending loop of Henle; MC, mesangial cells; Podo, podocytes. **F** Fraction of reads covering intronic variants in each of the three profiles

In order to better understand the effects of background noise, it is helpful to understand its origins and composition. To this end, we constructed profiles representing endogenous, contaminating and ambient expression profiles by using *M. m. domesticus* allele counts in *M. m. domesticus* cells (endogenous), *M. m. domesticus* allele counts in *M. m. castaneus* cells (contamination) and *M. m. domesticus* allele counts in empty droplets (empty) (Fig. 3A , B; Additional file 1: Fig. S5A-E).

The number of contaminating UMI counts per cell is at a similar level as the UMI counts in empty droplets in all replicates (Fig. 3C, Additional file 1: Fig. S5F). Moreover, if the median UMI count in empty droplets is high for one replicate, we also observe more contaminating UMIs, which is also consistent with ambient RNA as the main source for background noise.

In addition, when comparing pseudobulk aggregates of the three scRNA-seq replicates, we find that the contamination profiles correlate highly and similarly well with empty (Spearman’s $\rho =0.73-0.85$) and endogenous profiles (Spearman’s $\rho =0.70-0.87$), while for the nuc2 and nuc3 the contamination profiles are clearly more similar to the empty (Spearman’s $\rho \sim 0.85$) than to the endogenous profiles (Spearman’s $\rho \sim 0.50$) (Fig. 3B).

Using deconvolution analysis[16], we reconstructed the cell type composition from the pseudobulk profiles. In agreement with the correlation analysis, we find that in our scRNA-seq data the cell type compositions inferred for endogenous, contamination and empty counts are by and large similar with a slight increase in the PT-profile in empty droplets, suggesting that this cell type is more vulnerable to dissociation procedure than other cell types. In contrast, deconvolution of the empty droplet and contamination fraction of nuc2 and nuc3, that in contrast to the scRNA-seq data were prepared from frozen samples, shows a clear shift in cell type composition with a decreased PT fraction (Fig. 3C, Additional file 1: Fig. S6).

Moreover, we expect that cytosolic mRNA contributes more to the contaminating profile than to the endogenous profile. Indeed, in our snRNA-seq data we find that in good nuclei (endogenous molecules) more than 25% of the allele counts fall within introns, while out of the molecules from empty droplets less than 18% fall within introns (Fig. 3D). Similarly also in the scRNA-seq data, we find with $\sim 14\%$ more intron variants than in empty droplets. The intron fraction of the contaminating molecules lies in-between the endogenous and the empty droplet fraction, but is in all cases much closer to the empty intron fraction, thus suggesting again that the majority of the background noise likely originates from ambient RNA.

### Only little evidence for barcode swapping

In addition to ambient RNA, barcode swapping resulting from chimera formation during PCR amplification can also contribute to background noise. With the 12bp UMIs from 10x, the probability that we capture the same UMI-cell barcode combination twice independently is very low, hence how often we find the same combination of cell barcode and UMI associated with more than one gene is a good measure for barcode swapping [7]. The median fraction of such chimeric molecules varies between 0.2% for rep3 and 0.7% for nuc3 (Additional file 1: Fig. S7A). In line with our expectations outlined before, the absolute amount of swapping per cell correlates strongly with the total molecule count (Additional file 1: Table S1). In combination with the weak correlation between the number of contaminating with endogenous molecule counts, this supports the notion that the majority of background noise does not come from swapping. To be more quantitative, we combine the swapping and the total background fractions to estimate how much swapping could contribute to the total background and find that the median contribution of barcode swapping to background noise is lower than 10% for all replicates (Additional file 1: Fig. S7B).

Furthermore, molecules with a swapped barcode are expected to have a lower average number of reads per UMI. This is because chimera that are formed late during PCR subsequently undergo less amplification [7]. Thus, if the majority of contaminating reads were to originate from barcode swapping, we would expect that the distribution of reads per UMI for cross-genotype contaminating molecules (cont) is similar to that of observed chimeras. This is not what we see (Additional file 1: Fig. S7C): The distribution of reads per UMI for contaminating reads is much more distinct from the distribution for chimeras (Kolmogorov-Smirnov distance, $\Delta _n = 0.381$ (rep3) to 0.595 (nuc3)) than for endogenous reads ($\Delta _n = 0.008$ (rep2) to 0.046 (rep3)). In summary, we find that barcode swapping during library preparation only contributes little to the overall background noise in this data.

### The impact of contamination on marker gene analyses

**Fig. 4:**
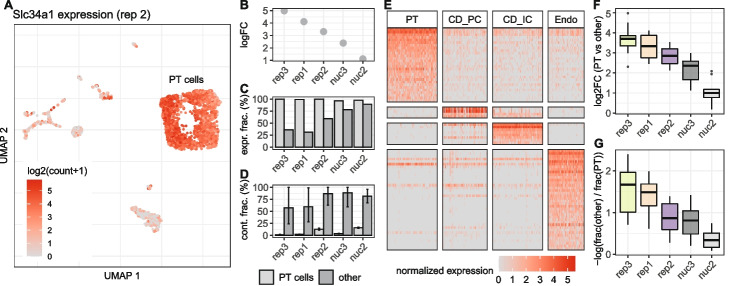
Background noise affects differential expression and specificity of cell type specific marker genes. **A** UMAP representation of replicate 2 colored by the expression of Slc34a1, a marker gene for cells of the proximal tubule (PT). Besides high counts in a cluster of PT cells, Slc34a1 is also detected in other cell type clusters. Differential expression analysis between PT and all other cells shows a decrease of the detected log fold change of Slc34a1 (**B**) at higher background noise levels, as well as an increase of the fraction of non PT cells in which UMI counts of Slc34a1 were detected (**C**). **D** Estimation of the background noise fraction of Slc34a1 expression indicates that the majority of counts in non PT cells originates from background noise. Error bars indicate 90% profile likelihood confidence intervals. **E** Heatmap of marker gene expression for four cell types in replicate 2, downsampled to a maximum of 100 cells per cell type. **F** Comparison across replicates of log2 fold changes of 10 PT marker genes calculated based on the mean expression in PT cells against mean expression in all other cells. **G** For the same set of genes as in **E**, the log ratio of fraction of cells in which a gene was detected in others and PT cells shows how specific the gene is for PT cells

The ability to distinguish hitherto unknown cell types and states is one of the greatest achievements made possible by single cell transcriptome analyses. To this end, marker genes are commonly used to annotate cell clusters for which available classifications appear insufficient. An ideal marker gene would be expressed in all cells of one type but in none of the other present cell types. Thus, when comparing expression levels of one cell type versus all others, we expect high log2-fold changes, the higher the change the more reliable the marker. However, such a reliance on marker genes also makes this type of analysis vulnerable to background noise. Our whole kidney data can illustrate this problem well, because with the very frequent proximal tubular (PT) cells we have a dominant cell type for which rather specific marker genes are known [17]. Slc34a1 encodes a phosphate transporter that is known to be expressed exclusively in PT cells [18, 19]. As expected, it is expressed highly in PT cells, but it is also present in a high fraction of other cells (Fig. 4A, E; Additional file 1: Fig. S8). Moreover, the log2-fold changes of Slc34a1 are smaller in replicates with larger background noise, indicating that the detection of Slc34a1 in non-PT cells is likely due to contamination (Fig. 4B–D). We observe the same pattern for other marker genes as well: they are detected across all cell types (Fig. 4E, Additional file 1: Fig. S9) and an increase of background noise levels goes along with decreasing log2-fold changes and increasing detection rates in other cell types (Fig. 4F,G). Thus, the power to accurately detect marker genes decreases in the presence of background noise.

### Benchmark of background noise estimation tools

**Fig. 5:**
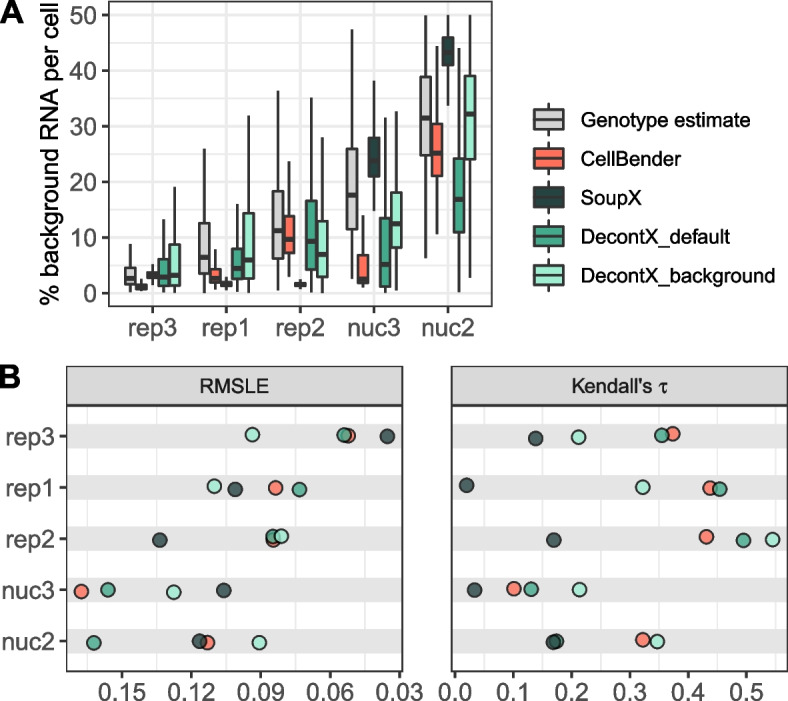
Accuracy of computational background noise estimation. **A** Estimated background noise levels per cell based on genetic variants (gray) and different computational tools. **B** Taking the genotype-based estimates as ground truth, Root Mean Squared Logarithmic Error (RMSLE) and Kendall rank correlation serve as evaluation metrics for cell-wise background noise estimates of different methods. Low RMSLE values indicate high similarity between estimated values and the assumed ground truth. High values of Kendall’s $\tau$ correspond to good representation of cell to cell variability in the estimated values

Given that background noise will be present to varying degrees in almost all scRNA-seq and snRNA-seq replicates, the question is whether background removal methods can alleviate the problem without the information from genetic variants. SoupX [11], DecontX [16] and CellBender [4], all provide an estimate of the background noise level per cell. Here, we use our genotype-based background estimates as ground truth to compare it to the estimates of the three background removal methods (Fig. 5A, Additional file 1: Fig. S10). All methods have adjustable parameters, but also provide a set of defaults. For CellBender the user can adjust the nominal false positive rate to put a cap on losing information from true counts. For SoupX and DecontX the resolution of the clustering of cells that is later used to model the endogenous counts can be adjusted. In addition, SoupX can be provided with an expected background level and for DecontX the user can provide a custom background profile rather than using the default estimation strategy for the background profile. At least with our reference dataset, CellBender does not seem to profit from changing the defaults, while SoupX’s performance is boosted, if provided with realistic background levels (Additional file 1: Fig. S15). Because in a real case scenario, the true background level is unknown, we decided to report the SoupX performance metrics under default settings. DecontX defaults to estimating the putative background profile from averaging across intact cells. To ensure comparability, we report DecontX’s performance with empty droplets as background profile (DecontX$_{background}$) in addition to DecontX with default settings (DecontX$_{default}$).

We find that CellBender and DecontX can estimate background noise levels similarly well for the scRNA-seq replicates, while SoupX tends to underestimate background levels and also cannot capture the cell to cell variation as measured by the correlation with the ground truth (Fig. 5B). For nuc2 and nuc3 , SoupX performs better at estimating global background levels, but as for the scRNA-seq still cannot capture cell to cell variation. In contrast, both CellBender and DecontX perform worse for nuc2 and nuc3. Moreover for nuc2 and nuc3, DecontX with default setting provides worse estimates than with empty droplets as background profile.

All in all, CellBender shows the most robust performance across replicates with default settings, while DecontX’ and SoupX’ performance seems to require parameter tuning. A drawback of CellBender is its runtime. While SoupX and DecontX take seconds and minutes to process one 10× channel, CellBender takes $\sim 45$ CPU hours. However, parallelization is possible.

All methods struggled most with the nuc3 replicate that has the fewest *M. m. castaneus* cells and the lowest cell type diversity among our five data sets (Fig. 1B, E). This also presents a problem for other downstream analyses and thus we do not consider nuc3 further.

### Effect of background noise removal on marker gene detection

**Fig. 6:**
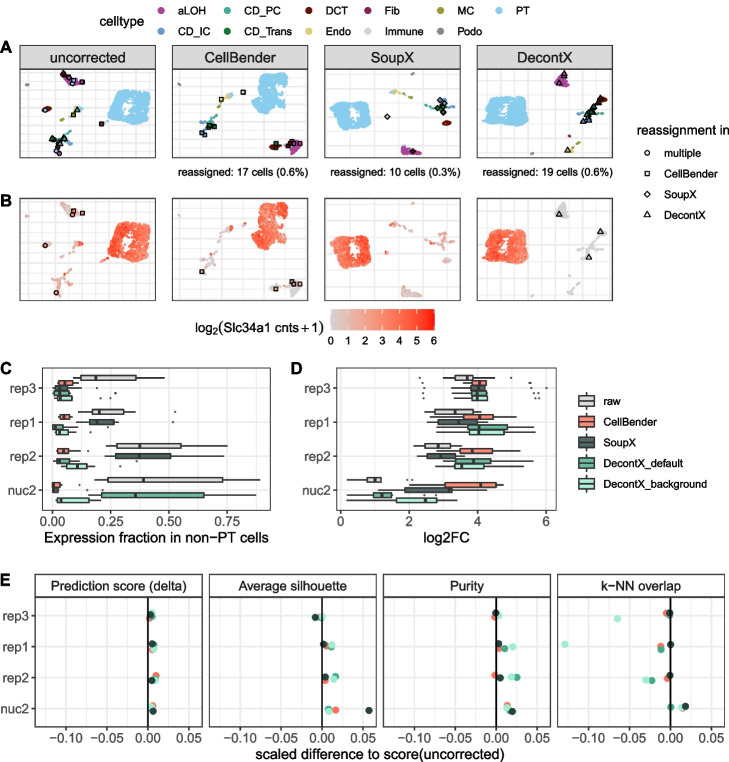
Effect of background removal on downstream analysis. **A** UMAP representation of replicate 2 single-cell data before and after background noise correction, colored by cell type labels obtained from reference based classification. Individual cells that received a new label after correction are highlighted. PT, proximal tubule; CD_IC, intercalated cells of collecting duct; CD_PC, principal cells of collecting duct; CD_Trans, transitional cells of collecting duct; CNT, connecting tubule; DCT, distal convoluted tubule; Endo, endothelial; Fib, fibroblasts; aLOH, ascending loop of Henle; dLOH, descending loop of Henle; MC, mesangial cells; Podo, podocytes. **B** Expression of the PT cell marker Slc34a1 before and after background noise correction in replicate 2. Cells that were classified as PT cells in the uncorrected data, but got reassigned after correction, are highlighted. **C**, **D** Differential expression analysis of 10 PT markers, evaluating the expression fraction in non-PT cells (**C**) and the log2 fold change between PT and all other cells (**D**). **E** Evaluation metrics for the effect of background noise correction on classification and clustering. For each metric the change relative to the uncorrected data is depicted. The values were scaled by the possible range of each metric. Prediction score: cell-wise score “delta” of reference based classification with SingleR [20]. Average silhouette: Mean of silhouette widths per cell type. Purity: Cluster purity calculated on cell type labels as ground truth and Louvain clusters as test labels. *k*-NN overlap: overlap of the *k*=50 nearest neighbors per cell compared to genotype-cleaned reference *k*-NN graph

Above we have shown that computational methods can estimate background noise levels per cell. Moreover, all three methods provide the user with a background corrected count matrix for downstream analysis. Here, we compare the outcomes of marker gene detection, clustering and classification when using corrected count matrices from SoupX, DecontX, and CellBender (Fig. 6A, Additional file 1: Fig. S11). To characterize the impact on marker gene detection, we first check in how many cells an unexpected marker gene was detected; for example, how often Slc34a1 was detected in cells other than PTs (Fig. 6B). Without correction we find Slc34a1 reads in $\sim 60\%$ of non-PT cells of rep2, SoupX reduces this rate to 54%, CellBender to 7% and DecontX$_{background}$ to 9%. DecontX$_{default}$ manages to remove most contaminating reads reducing the Slc34a1 detection rate outside PTs to 2%. While we find a similar ranking when averaging across several marker genes from the PanglaoDB database [17] and scRNA-seq replicates (Fig. 6C), the ranking changes for nuc2: DecontX$_{default}$ fails: after correction, Slc34a1 is still found in 87% of non-PT cells while DecontX$_{background}$ is better with a rate of 20%. Here, CellBender and SoupX are clearly better with reducing the Slc34a1 detection rate to 4% and $<1\%$, respectively (Additional file 1: Fig. S12).

Even though the changes in the marker gene detection rates outside the designated cell type seem dramatic (Additional file 1: Fig. S13A), the identification of marker genes [21] is affected only a little. CellBender correction has the largest effect on marker gene detection, yet 8 from the top 10 genes without correction remain marker genes with CellBender correction (Spearman’s correlation for top 100 $\rho =0.84$). In contrast, in the nuc2 data with high background levels, the change in marker gene detection is dramatic. Here, only one of the top 10 marker genes remains after correction (Spearman’s correlation for top 100 $\rho =0.04$). The largest improvement is achieved with CellBender: After correction, four out of the top 10 were known marker genes [17], while this overlap amounted to only one in the raw data (Additional file 1: Fig. S13B). Moreover, we find that background removal also increases the detected log-fold-changes of known marker genes across all replicates and methods, with CellBender providing the largest improvement (Fig. 6D, Additional file 1: Fig. S13C).

### Effect of background noise removal on classification and clustering

One of the first and most important tasks in single cell analysis is the classification of cell types. As described above, we could identify 13 cell types in our uncorrected data using an external single cell reference dataset [14, 20]. Going through the same classification procedure after correction for background noise, changes the classification of only very few cells (Fig. 6A, Additional file 1: Fig. S11). For the scRNA-seq experiments $<1\%$ and for the nuc2 up to 1.3% of cells change labels after background removal compared to the classification using raw data. Before correction, these cells are mostly located in clusters dominated by a different cell type (Fig. 6A). Moreover, these cells tend to have higher background levels as exemplified by the PT-marker gene Slc34a1 (Fig. 6B). Finally, background removal — irrespective of the method - improves the classification prediction scores (Fig. 6E, Additional file 1: Fig. S14). Together, this indicates that background removal improves cell type classification.

Similarly, background removal also results in more distinct clusters. Here, we reason that cells of the same cell type should cluster together and evaluate the impact of background removal (1) on the silhouette scores for cell types and (2) on the cell type purity of each cluster using unsupervised clustering (Fig. 6E). For the scRNA-seq data DecontX results in the purest and most distinct clusters, while for the nuc2 data SoupX wins in these categories.

All in all, it seems clear that all background removal methods sharpen the broad structure of the data a little, but how about fine structure? To answer this question, we turn again to the genotype cleaned data to obtain a ground truth for the *k*-nearest neighbors of a cell and calculate how much higher the overlap of the background corrected data is with this ground truth as compared to using the raw data (Fig. 6E). For the scRNA-seq data, DecontX has the largest improvement on the broad structure, but at same time in particular DecontX$_{background}$ lowers the overlap in *k*-NN with our assumed ground truth, suggesting that this change in structure is a distortion rather than an improvement. SoupX leaves the fine structure by and large unchanged in the scRNA-seq data, while both CellBender and DecontX make the fine structure slightly worse. In contrast, for the high background levels of the nuc2, all background removal methods achieve an improvement, with SoupX and CellBender performing best.

## Discussion

Here we provide a dataset for the characterization of background noise in 10× Genomics data that is ideal to benchmark background removal methods. The mixture of cell types in our kidney data provides us with realistic cell type diversity and the mixture of mouse subspecies enables us to identify foreign alleles in a cell, thus resulting in a dataset that allows us to quantify background noise across diverse cell types and features. In addition, the replicates exhibit varying degrees of contamination, enabling us to evaluate the effects of low, intermediate, and high background levels. Given that every sample poses new challenges for the preparation of a suspension of intact cells or nuclei that is needed for a 10× experiment, we expect that such variability in sample quality is not unusual. Consequently, marker gene identification is affected and markers appear less specific, as they are detected in cell types where they are not expressed. The degree of this issue directly depends on background noise levels (Fig. 4). This particular problem has been observed previously and has been used as a premise to develop background correction methods [4, 11, 22].

The novelty of this analysis is that — thanks to the mix of mouse subspecies — we are able to obtain expression profiles that describe the source of contamination in each sample and also have a ground truth for a more realistic dataset. We started to characterize background noise by comparing the contamination profile with the profile of empty droplets and that of endogenous counts of good cells. In agreement with the idea that ambient RNA is due to leakage of cytosol, we find that empty droplets show less evidence for unspliced mRNA molecules and that the unspliced fraction in the contamination profiles is similar to that of empty droplets. This is a first hint that a large proportion of the background noise is ambient RNA. In addition, we find only little direct evidence for barcode swapping as provided by chimeric UMIs, which only explains up to 10% of background noise (Additional file 1: Fig. S7B). Hence, also the observed correlation between cell size and the absolute amounts of background noise per cell in most of the replicates is likely due to variation in dropout rates [4] (Fig. 2B, Additional file 1: Table S1).

Another important insight from comparing contamination, empty and endogenous profiles is that we can deduce the origin of the contamination. While for rep1-3 all three profiles are highly correlated and are the result of very similar cell type mixtures, for nuc2 and nuc3 the empty and the contamination profiles are distinct from the expected endogenous mixture profile. Encouragingly the endogenous profiles of all replicates agree well with one another as well as with the cell type proportions from the literature [14, 23]. Moreover, the higher similarity of the contamination to the empty than to the endogenous profile supports the notion that the majority of background noise is ambient RNA and hence using the empty rather than the endogenous profile as a reference to model background noise is the better choice for our data. Indeed, the performance of DecontX for nuc2 is improved by providing the empty droplet profile as compared to the endogenous profile which is the default (Fig. 5A). We also observed that SoupX performs much better for the snRNA-seq data than the scRNA-seq data. We speculate that the marker gene identification that is the basis for estimating the experiment-wide average contamination is hampered by the fact that our dataset has one very dominant cell type that has the same prevalence in the empty droplets, thus masking all background. However, even if SoupX gets the overall background levels right, it by design grossly underestimates the variance among cells and cannot capture the cell to cell variation (Fig. 5B, C). Overall CellBender provides the most accurate estimates of the background noise levels and also captures the cell to cell variation rather well. We note that this finding is largely due to the robustness of CellBender to cell type composition and the source of contamination, that determines the similarity between the contamination and the endogenous profiles.

In line with this, also marker gene detection is most improved by CellBender, which is the only method that removes marker gene molecules from other cell types and increases the log-fold-change consistently well. The effect of background removal on other downstream analyses is much more subtle. For starters, classification using an external reference is rather robust. Even with high levels of background noise, background removal improves classification only for a handful of cells and we cannot say that one method outperforms the others (Fig. 6E, Additional file 1: Fig. S14). Similarly, the broad structure of the data improves only minimally and this minimal improvement comes at the cost of disrupting fine structure (Fig. 6E). Here, again CellBender strikes the best balance between removing variation but preserving the fine structure, while DecontX tends to remove too much within-cluster variability, as the *k*-NN overlap with the genotype-based ground truth for DecontX is even lower than for the raw data. All in all, CellBender shows the best performance in removing background noise.

## Conclusions

Levels of background noise can be highly variable within and between replicates and the contamination profiles do not always reflect the cell type proportions of the sample. Marker gene detection is affected most by this issue, in that known cell type specific marker genes can be detected in cell clusters where they do not belong. Existing methods for background removal are good at removing such stray marker gene molecule counts. In contrast, classification and clustering of cells is rather robust even at high levels of background noise. Consequently, background removal improves the classification of only few cells. Moreover, it seems that for low and moderate background levels the tightening of existing broad structures may go at the cost of fine structure. In summary, for marker gene analysis, we would always recommend background removal, but for classification, clustering and pseudotime analyses, we would only recommend background removal when background noise levels are high.

## Methods

### Mice

Three mouse strains were ordered from Jackson Laboratory at 6–8 weeks of age: C57BL/6J (000664), CAST/EiJ (000928), and 129S1/SvlmJ (002448). All animals were subjected to intracardiac perfusion of PBS to remove blood. Kidneys were dissected, divided into 1/4s, and subjected to the tissue dissociation protocol, stored in RNAlater, or snap-frozen in liquid nitrogen.

### Tissue dissociation for single cell isolation

The single cell suspensions were prepared following an established protocol [24] with minor modifications. In detail, one of each kidney sagittal quarter from three perfused mice of different strains C57BL/6, CAST/EiJ and 129S1/SvImJ were harvested into cold RPMI (Thermo Fisher Scientific, 11875093) with 2% heat-inactivated Fetal Bovine Serum (Gibco, Thermo Fisher Scientific, 16140-071; FBS) and 1% penicillin/streptomycin (Gibco, Thermo Fisher Scientific, 15140122). Each piece of the tissue was then minced for 2 min with a razor blade in 0.5 ml 1x liberase TH dissociation medium (10x concentrated solution from Millipore Sigma, 05401135001, reconstituted in DMEM/F12(Gibco, Thermo Fisher Scientific, 11320-033 in a petri dish on ice. The chopped tissue pieces were then pooled into one 1.5 ml Eppendorf tube and incubated in a thermomixer at 37${}^{\circ }$C for 1 hour at 600rpm with gentle pipetting for trituration every 10 min. The digestion mix was then transferred to a 15 ml conical tube and mixed with 10 ml 10% FBS RPMI. After centrifugation in a swinging bucket rotor at 500g for 5 min at 4 °C and supernatant removal, the pellet was resuspended in 1ml red blood cell lysing buffer (Sigma Aldrich, R7757). The suspension was spun down at 500g for 5 min at 4 °C followed by supernatant removal. The pellet cleared of the red blood cell ring was then resuspended in 250 $\upmu$l Accumax (Stemcell Technologies, 7921) and incubated at 37 °C for 3 mins. The reaction was stopped by mixing with 5 ml 10% FBS RPMI and spinning down at 500g for 5 min at 4 °C followed by supernatant removal. The cell pellet was then resuspended in PBS with 0.4% BSA (Sigma, B8667) and passed through a 30 $\upmu$m filter (Sysmex, 04-004-2326). The cell suspension was then assessed for viability and concentration using the K2 Cellometer (Nexcelom Bioscience) with the AOPIcell stain (Nexcelom Bioscience, CS2-0106-5ML).

### Nuclei isolation from RNAlater preserved frozen tissue

The single nuclei suspensions were prepared following an established protocol [25] with minor modifications. In detail, the RNAlater reserved frozen tissue of 3 mice kidney quarters were thawed and transferred to one petri dish preloaded with 1 ml TST buffer containing 10 mM Tris, 146 mM NaCl, 1 mM CaCl2, 21 mM MgCl2, 0.03% Tween-20 (Roche, 11332465001), and 0.01% BSA (Sigma, B8667). It was minced with a razor blade for 10 min on ice. The homogenized tissue was then passed through a 40 $\upmu$m cell strainer (VWR, 21008-949) into a 50 ml conical tube. One ml TST buffer was used to rinse the petri dish and collect the remaining tissue into the same tube. It was then mixed with 3 ml of ST buffer containing 10 mM Tris, 146 mM NaCl, 1 mM CaCl2, and 21 mM MgCl2 and spun down at 500g for 5 min at 4 °C followed by supernatant removal. In the second experiment this washing step was repeated 2 more times. The pellet was resuspended in 100 $\upmu$l ST buffer and passed through a 35 $\upmu$m filter. The nuclei concentration was measured using the K2 Cellometer (Nexcelom Bioscience) with the AO nuclei stain (Nexcelom Bioscience, CS1-0108-5ML).

### Single-cell and single-nucleus RNA-seq

The cells or nuclei were loaded onto a 10× Chromium Next GEM G chip (10x Genomics, 1000120) aiming for recovery of 10,000 cells or nuclei. The RNA-seq libraries were prepared using the Chromium Next GEM Single Cell 3’ Reagent kit v3.1 (10× Genomics, 1000121) following vendor protocols. The libraries were pooled and sequenced on NovaSeq S1 100c flow cells (Illumina) with 28 bases for read1, 55 bases for read2 and 8 bases for index1 and aiming for 20,000 reads per cell.

### Processing and annotation of scRNA-seq and snRNA-seq data

The scRNA-seq and snRNA-seq data were processed using Cell Ranger 3.0.2 using as reference genome and annotation mm10 version 2020A for the scRNA-seq data and and a pre-mRNA version of mm10 2.1.0 as reference for snRNA-seq. In order to identify cell containing droplets we processed the raw UMI matrices with the DropletUtils package [5]. The function barcodeRanks was used to identify the inflection point on the total UMI curve and the union of barcodes with a total UMI count above the inflection point and Cell Ranger cell call were defined as cells.

For cell type assignment we used 3 scRNA-seq and 4 snRNA-seq experiments from Denisenko et al. [14] as a reference. Cells labeled as “Unknown” (*n*=46), “Neut” (*n*=17) and “Tub” (*n*=1) were removed. The reference was log-normalized and split into seven count matrices based on chemistry, preservation and dissociation protocol. Subsequently, a multi-reference classifier was trained using the function *trainSingleR* with default parameters of the R package SingleR version 1.8.1 [20]. After this processing, we could use the data to classify our log-normalized data using the *classifySingleR* function without fine-tuning (fine.tune = F). Hereby, each cell is compared to all seven references and the label from the highest-scoring reference is assigned. Some cell type labels were merged into broader categories after classification: cells annotated as “CD_IC,” “CD_IC_A,” or “CD_IC_B” were relabeled as “CD_IC,” cells annotated as “T,” “NK,” “B,” or “MPH” were relabeled as “Immune.” Cells that were unassigned after pruning of assignments based on classification scores were removed for subsequent analyses.

### Demultiplexing of mouse strains

A list of genetic variants between mouse strains was downloaded in VCF format from the Mouse Genomes Project [13], accessed on 21 October 2020. This reference VCF file was filtered for samples CAST_EiJ, C57BL_6NJ and 129S1_SvImJ and chromosomes 1–19. Genotyping of single barcodes was performed with cellsnp-lite [26], filtering for positions in the reference VCF with a coverage of at least 20 UMIs and a minor allele frequency of at least 0.1 in the data (–minCOUNT 20, –minMAF 0.1). Vireo [22] was used to demultiplex and label cells based on their genotypes. Only cells that could be unambiguously assigned to CAST_EiJ (CAST), C57BL_6NJ (BL6) or 129S1_SvImJ (SvImJ) were kept, cells labeled as doublet or unassigned were removed.

### Genotype-based estimation of background noise

Based on the coverage filtered VCF-file (see above), we identified homozygous SNPs that distinguish the three strains and removed SNPs that had predominantly coverage in only one of the strains (1st percentile of allele frequency).

In most parts of the analysis, we focused on the comparison between the mouse subspecies, *M. m. domesticus* and *M. m. castaneus*. To this end, we subseted reads (UMI-counts) that overlap with SNPs that distinguish the two mouse subspecies.

To estimate background noise levels based on allele counts of genetic variants, an approach described in Heaton et al.[15] was adapted to estimate the total amount of background noise for each cells. First, the abundance of endogenous and foreign allele counts (i.e., cross-genotype background noise) was quantified per cell. Because of the filter for homozygous variants, there are two possible genotypes for each locus, denoted as 0 for the endogenous allele, i.e., the expected allele based on the strain assignment of the cell, and 1 for the foreign allele. The probability for observable background noise at each locus *l* in cell *c* is given by

1 $$\begin{aligned} p=\rho _{c}*\frac{A_{l,1}}{A_{l,0}+A_{l,1}} \end{aligned}$$

where $\rho _{c}$ is the total background noise fraction in a cell and the experiment wide (over cells and empty droplets) foreign allele fraction is calculated from the foreign allele counts $A_{l,1}$ and the endogenous allele counts $A_{l,0}$. The foreign allele fraction is then used to account for intra-genotype background noise (contamination within endogenous allele counts).

The observed allele counts $A_c$ per cell are modeled as draws from a binomial distribution with the likelihood function:

2 $$\begin{aligned} P(A_c|\rho _{c}) = \prod _{l \in L}{A_{l,c,0}+A_{l,c,1}\atopwithdelims (){A_{l,c,1}}}p^{A_{l,1}}(1-p)^{A_{l,0}} \end{aligned}$$

A maximum likelihood estimate of $\rho _c$ was obtained using one dimensional optimization in the interval [0,1].

The 95% confidence interval of each $\rho _c$ estimate was calculated as the profile likelihood using the function *uniroot* of the R package stats [27].

### Comparison of endogenous, contamination, and empty droplet profiles

Empty droplets were defined based on the UMI curve of the barcodes ranked by UMI counts, thus selecting barcodes from a plateau with $\sim 500-1000$ UMIs (Additional file 1: Fig. S5). For the following analysis, the presence of *M. m. domesticus* alleles in *M. m. domesticus* cells (i.e., endogenous), in *M. m. castaneus* cells (i.e., contamination) and empty droplets was compared. After this filtering, we summarized counts per gene and across barcodes of the same category to generate pseudobulk profiles.

In order to estimate cell type composition in the empty and contamination profiles, we used the deconvolution method implemented in SCDC[16], the endogenous single cell allele counts from the respective replicate were used as reference (*qcthreshold = *0.6). In addition, cell type filtering (frequency>0.75%) was applied. Endogenous, contamination and empty pseudobulk profiles from each replicate were deconvoluted using their respective single cell/single nucleus reference.

To compare the correlation between the different profiles, pseudobulk counts were downsampled to the same total size.

### Detection of barcode swapping events

Information about the number of reads per molecule and the combination of cell barcode (CB), UMI and gene were extracted from the molecule info file in the Cellranger output. We assume that a combination of CB and UMI corresponds to a single original molecule. Thus we define a PCR chimera as a non-unique CB-UMI combination in which multiple genes were associated with the same CB and UMI. Since we can only detect PCR chimera, if we detect at least 2 reads for a CB-UMI combination, we also restrict the total molecule count to CB-UMI combinations with at least 2 reads for the calculation of the chimera fraction.

For the comparison of reads/UMI the identified chimera were intersected with identified cross-genotype contamination. To this end, the the analysis was restricted to *M. m. castaneus* cells and CB-UMI-gene combinations which can be associated with an informative SNP. The number of reads/UMI was summarized per CB-UMI-gene combination for chimera (as defined above), unique CB-UMI-gene combinations with coverage for an endogenous allele (endo) and unique CB-UMI-gene combinations with coverage for a foreign allele (cont).

### Evaluation of marker gene expression

A list of marker genes for Proximal tubule cells (PT), Principal cells (CD_PC), Intercalated cells (CD_IC), and Endothelial cells (Endo) was downloaded from the public database PanglaoDB [17], accessed on 13 May 2022.

Log2 fold changes contrasting PT cells against all other cells were calculated with Seurat using the function *FindMarkers* after normalization with *NormalizeData*. The expression fraction *e* of PT markers was calculated as the fraction of cells for which at least 1 count of that gene was detected. To contrast expression fraction in PT cells against non-PT, the negative log-ratio was calculated as $-log((e_{PT}+1)/(e_{non-PT}+1))$.

### Computational background noise estimation and correction methods

*CellBender *[4] makes use of a deep generative model to include various potential sources of background noise. Cell states are encoded in a lower-dimensional space and an integer matrix of noise counts is inferred, which is subsequently subtracted from the input count matrix to generate a corrected matrix.

The *remove-background* module of CellBender v0.2.0 was run on the raw feature barcode matrix as input, with a default *fpr* value of 0.01. For the comparison of different parameter settings, *fpr* values of 0.05 and 0.1 were also included in the analysis. For the parameter *expected-cells* the number of cells after cell calling and filtering in each replicate was provided. The parameter *total-droplets-included* was set to 25,000.

*SoupX *[11] estimates the experiment-wide amount of background noise based on the expression of strong marker genes that are expected to be expressed exclusively in one cell type. These genes can either be provided by the user or identified from the data. A profile of background noise is inferred from empty droplets. This profile is subsequently removed from each cell after aggregation into clusters to generate a corrected count matrix.

Cluster labels for SoupX were generated by Louvain clustering on 30 principal components and a resolution of 1 as implemented by *FindClusters* in Seurat after normalization and feature selection of 5000 genes. Providing the CellRanger output and cluster labels as input, data were imported into SoupX version 1.6.1 and the background noise profile was inferred with *load10X*. The contamination fraction was estimated using *autoEstCont* and background noise was removed using *adjustCounts* with default parameters.

For the comparison of parameter settings, different resolution values (0.5, 1, 2) for Louvain clustering were tested, alongside with manually specifying the contamination fraction (0.1, 0.2).

*DecontX *[8] is a Bayesian method that estimates and removes background noise by modeling the expression in each cell as a mixture of multinomial distributions, one native distribution cell’s population and one contamination distribution from all other cell populations. The main inputs are a filtered count matrix only containing barcodes that were called as cells and a vector of cluster labels. The contamination distribution is inferred as a weighted combination of multiple cell populations. Alternatively, it is also possible to obtain an empirical estimation of the contamination distribution from empty droplets in cases where the background noise is expected to differ from the profile of filtered cells.

The function *decontX* from the R package celda version 1.12.0 was run on the filtered, unnormalized count matrix and clusters were inferred with the implemented default method based on UMAP dimensionality reduction and dbscan [28] clustering. For the “DecontX_default” results the parameter “background” was set to NULL, i.e., estimating background noise based on cell populations in the filtered data only. “DecontX_background” results were obtained by providing an unfiltered count matrix including all detected barcodes as “background” to empirically estimate the contamination distribution. Besides the default clustering method implemented in DecontX, cluster labels obtained from Louvain clustering (resolution 0.5, 1, and 2) were also provided to test different parameter settings.

### Evaluation metrics

#### Estimation accuracy

The genotype-based estimates $\rho _c$ for *M. m. castaneus* cells served as ground truth to evaluate the estimation accuracy of different methods. For each method cell-wise background noise fractions $a_c$ were calculated from the corrected count matrix *X* and the uncorrected (“raw”) count matrix *R* as

3 $$\begin{aligned} a_c = 1-{\frac{\sum _gx_{c,g}}{\sum _gr_{c,g}}} \end{aligned}$$

for cells *c* and genes *g*.

**RMSLE **The Root Mean Squared Logarithmic Error (RMSLE) is a lower bound metric that we use to quantify the difference between estimated background noise fractions per cell $a_c$ from different computational background correction methods and the genotype-based estimates $\rho _c$, obtained from genotype based estimation. It is calculated as:

4 $$\begin{aligned} RMSLE = \sqrt {\frac{1}{n}\sum _{c=1}^{n}(log(a_c+1)-log(\rho _c+1))^2} \end{aligned}$$

**Kendall’s**

$\tau$ To evaluate how well cell-to-cell variation of the background noise fraction is captured by the estimated values $a_c$, the Kendall rank correlation coefficient $\tau$ to the genotype-based estimates $\rho _c$ was computed using the implementation in the R package stats [27] as $\tau = cor(a_c,\rho _c, method = ``kendall'')$.

#### Marker gene detection

The same set of 10 PT marker genes from PanglaoDB as in the “Evaluation of marker gene expression” section was used to evaluate the improvement on marker gene detection on corrected count matrices.

**Log2 fold change** for each gene between the average expression in PT cells and average expression in other cells were obtained using the *NormalizeData* and *FindMarkers* functions in Seurat version 4.1.1.

***Expression fraction*** Entries in each corrected count matrix were first rounded to the nearest integer. The expression fraction of each gene in a cell population was calculated as the fraction of cells for which at least 1 count of that gene was detected. For evaluation of PT marker genes, unspecific detection is defined as the expression fraction in non-PT cells.

#### Cell type identification

**Prediction score** Each corrected count matrix was log-normalized and reference-based classification in SingleR [20] was performed with a pre-trained model (see “Processing and annotation of scRNA-seq and snRNA-seq data” section) on data from Denisenko et al. [14]. SingleR provides *delta* values as a measure for classification confidence, which depicts the difference of the assignment score for the assigned label and the median score across all labels. The *delta* values for each cell were retrieved using the function *getDeltaFromMedian* relative to the cells highest-scoring reference. A prediction score per cell type was calculated by averaging *delta* values across individual cells and a global prediction score per replicate was calculated by averaging across cell type prediction scores.

**Average silhouette** The silhouette width is an internal cluster evaluation metric to contrast similarity within a cluster with similarity to the nearest cluster. The cell type annotations from reference-based classification were used as cluster labels here. Count matrices were filtered to select for *M. m. castaneus* cells and cell types with more than 10 cells. Distance matrices were computed on the first 30 principal components using euclidean distance as distance measure. Using the cell type labels and distance matrix as input, the average silhouette width per cell type was computed with the R package cluster version 2.1.4. An *Average silhouette* per replicate was calculated as the mean of cell type silhouette widths.

**Purity** Purity is an external cluster evaluation metric to evaluate how well a clustering recovers known classes. Here, *Purity* was used to assess to what extent unsupervised cluster labels correspond to cell types. Count matrices were filtered to select for *M. m. castaneus* cells and cell types with more than 10 cells and Louvain clustering as implemented in *FindClusters* of Seurat version 4.1.1 on the first 30 principal components and with a resolution parameter of 1 was used to get a cluster label for each cell. Providing cell type annotations as true labels alongside the cluster labels, *Purity* was computed with the R package ClusterR version 1.2.6 [29].

***k-NN overlap*** To evaluate the lower-dimensional structure in the data beyond clusters and cell-types *k*-NN overlap was used as described in Ahlmann-Eltze and Huber [30]. A ground truth reference *k*-NN graph was constructed on a ’genotype-cleaned’ count matrix, only counting molecules that carry a subspecies-endogenous allele. Raw and corrected count matrices were filtered to contain the same genes as in the reference and a query *k*-NN graph was computed on the first 30 principal components. The *k*-NN overlap summarizes the overlap of the 50 nearest neighbors of each cell in the query with the reference *k*-NN graph.

## Supplementary information

**Additional file 1: Supplementary Material.** This file contains Table S1 and Figures S1-15. **Table S1.** Spearman correlation analysis of background noise and barcode swapping. **Fig. S1.** Detection of cross-genotype contamination. **Fig. S2.** Estimation of background noise levels. **Fig. S3.** UMAP visualization showing the composition per replicate. **Fig. S4.** Definition of true cells and its effect on background noise estimates. **Fig. S5.** Definition of endogenous, empty droplet and contamination profiles across replicates. **Fig. S6.** Dissection of cell type contributions by deconvolution of pseudobulk profiles. **Fig. S7.** Identification of barcode swapping due to PCR chimeras. **Fig. S8.** Slc34a1 expression across replicates. **Fig. S9.** Expression of cell type marker genes. **Fig. S10.** Estimated background noise levels across cell types. **Fig. S11.** UMAP representations of all replicates before and after background noise correction. **Fig. S12.** Detected expression levels of Slc34a1 before and after background noise correction. **Fig. S13.** Effect of background noise correction on marker gene detection. **Fig. S14.** Evaluation metrics for cell type identification. **Fig. S15.** Evaluation of different parameter settings.

**Additional file 2.** Review history.

## Data Availability

The code used to analyse the data and benchmark the background methods is available on GitHub https://github.com/Hellmann-Lab/scRNA-seq_Contamination [31] under GPL-3.0 license and deposited in Zenodo under DOI 10.5281/zenodo.7941521 [32]. Larger files are available on a separate Zenodo repository [33]. All sequencing files were deposited in GEO under accession number GSE218853 [34].
