# Supplementary material for: The effect of background noise and its removal on the analysis of single-cell expression data
Source: Genome Biol. 2023 Jun 19;24:140. doi: 10.1186/s13059-023-02978-x (PMC10278251; doi:10.1186/s13059-023-02978-x)
Supplement: Supplementary file 1 — Additional file 1: Supplementary Material. This file contains Table S1 and Figures S1-15. Table S1. Spearman correlation analysis of background noise and barcode swapping. Fig. S1. Detection of cross-genotype contamination. Fig. S2. Estimation of background noise levels. Fig. S3. UMAP visualization showing the composition per replicate. Fig. S4. Definition of true cells and its effect on background noise estimates. Fig. S5. Definition of endogenous, empty droplet and contamination profiles across replicates. Fig. S6. Dissection of cell type contributions by deconvolution of pseudobulk profiles. Fig. S7. Identification of barcode swapping due to PCR chimeras. Fig. S8. Slc34a1 expression across replicates. Fig. S9. Expression of cell type marker genes. Fig. S10. Estimated background noise levels across cell types. Fig. S11. UMAP representations of all replicates before and after background noise correction. Fig. S12. Detected expression levels of Slc34a1 before and after background noise correction. Fig. S13. Effect of background noise correction on marker gene detection. Fig. S14. Evaluation metrics for cell type identification. Fig. S15. Evaluation of different parameter settings. [file 13059_2023_2978_MOESM1_ESM.pdf]

Supplementary Information

The effect of background noise and its removal on the analysis of single-cell expression data

Philipp Janssen[1] Zane Kliesmete[1], Beate Vieth[1], Xian Adiconis[2,3], Sean Simmons[2,3], Jamie Marshall[4], Cristin McCabe[2], Holger Heyn[5], Joshua Z. Levin[2,3], Wolfgang Enard[1], Ines Hellmann[1,*],

[1] Anthropology and Human Genomics, Department of Biology II, Ludwig-Maximilians Universitaet, Munich, Germany
[2] Klarman Cell Observatory, Broad Institute of Harvard and MIT, Cambridge, MA USA
[3] Stanley Center for Psychiatric Research, Broad Institute of Harvard and MIT, Cambridge, MA USA
[4] Broad Institute of Harvard and MIT, Cambridge, MA USA
[5] CNAG-CRG, Centre for Genomic Regulation, Barcelona Institute of Science and Technology, Barcelona, Spain

[*] correspondence hellmann@bio.lmu.de

Supplementary Tables

Table S1 Spearman correlation analysis of background noise and barcode swapping. Endogenous and contaminating allele counts refer to *M.m. castaneus* and *M.m. domesticus* allele counts in *M.m. castaneus* cells, respectively. Chimera refer to barcode swapping events that are observable by the association of multiple genes with the same cell barcode (CB)-UMI combination.

	Endogenous vs contaminating allele counts per cell		Chimera vs unique BC-UMI-gene counts per cell	
replicate	rho	p-value	rho	p-value
rep3	0.27	$<$2.2e-16	0.81	$<$2.2e-16
rep1	0.07	9e-08	0.81	$<$2.2e-16
rep2	0.06	0.0021	0.64	$<$2.2e-16
nuc3	0.03	0.499	0.77	$<$2.2e-16
nuc2	0.15	2.56e-09	0.52	$<$2.2e-16

Supplementary Figures

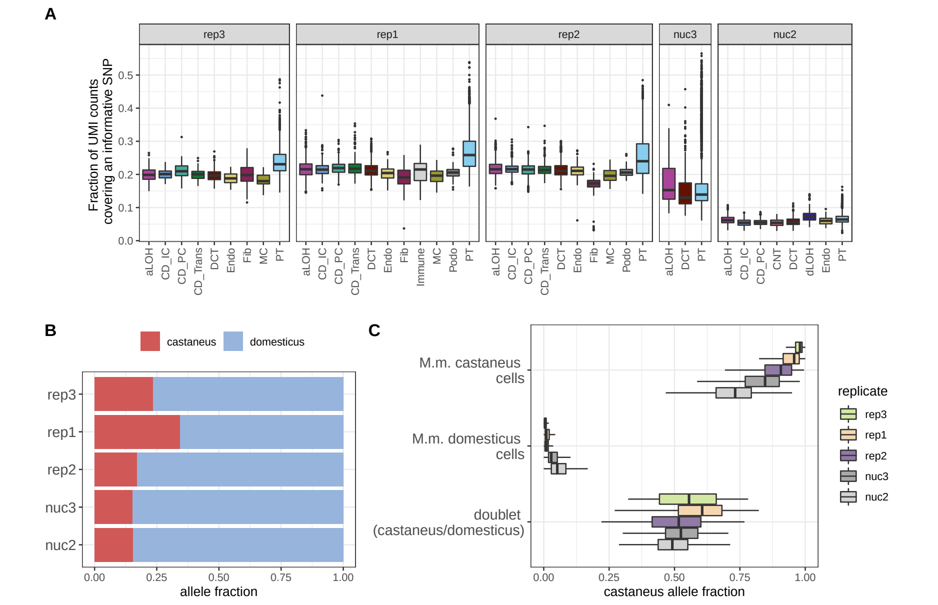

Fig. S1 Detection of cross-genotype contamination A) Variants that are variable between mouse subspecies were used to identify background noise. Boxplots show the fraction of detected molecules (UMI counts) that contain an informative SNP in each cell across cell types with more than 50 cells. CD_IC: intercalated cells of collecting duct; CD_PC: principal cells of collecting duct; CD_Trans: transitional cells of collecting duct; CNT: connecting tubule; DCT: distal convoluted tubule; Endo: endothelial; Fib: fibroblasts; aLOH: ascending loop of Henle; dLOH: descending loop of Henle; MC: mesangial cells; Podo: podocytes. B) For each informative SNP we can detect either the *M.m. castaneus* or the *M.m. domesticus* allele. The stacked bar plots indicate the allele fraction per replicate, integrating over all cells and SNPs. Since each replicate is a mixture experiment with a majority of *M.m. domesticus* cells, *M.m. domesticus* alleles are detected more often at covered informative SNPs. C) *M.m. castaneus* allele frequency per cell in cells from different subspecies and mixed-subspecies doublets. In all replicates varying amounts of *M.m. castaneus* alleles are detected in *M.m. domesticus* cells and vice versa, pointing towards background noise originating from cross-genotype contamination.

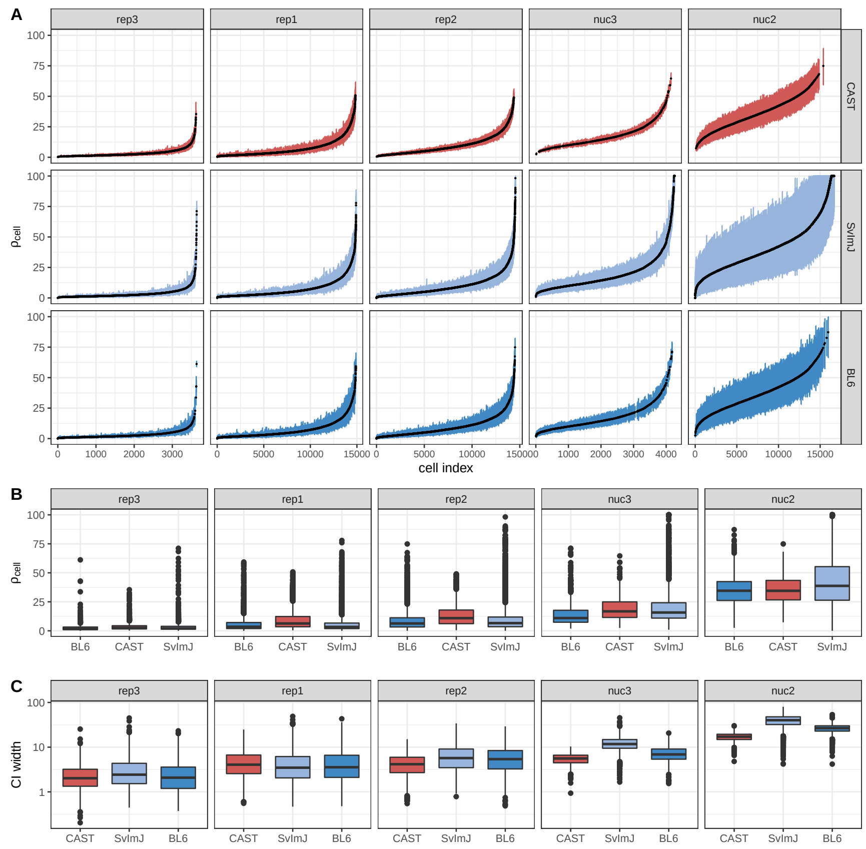

Fig. S2 Estimation of background noise levels. A) Estimates of background noise (ρ_{cell}) per cell. Cells were ordered by ascending ρ_{cell} in each replicate. Colored bars indicate 95% confidence intervals calculated by profile likelihood. B) Summary of ρ_{cell} estimates per strain. C) Width of confidence intervals for ρ_{cell}.

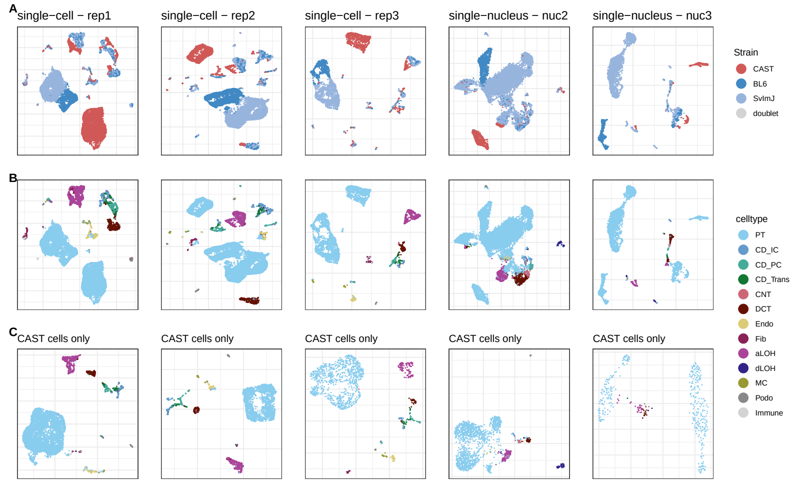

Fig. S3 UMAP visualization showing the composition per replicate of A) all cells, colored by strain assignment, B) all cells, colored by cell type assignment and C) *M. m. castaneus* cells only, colored by cell type assignment.

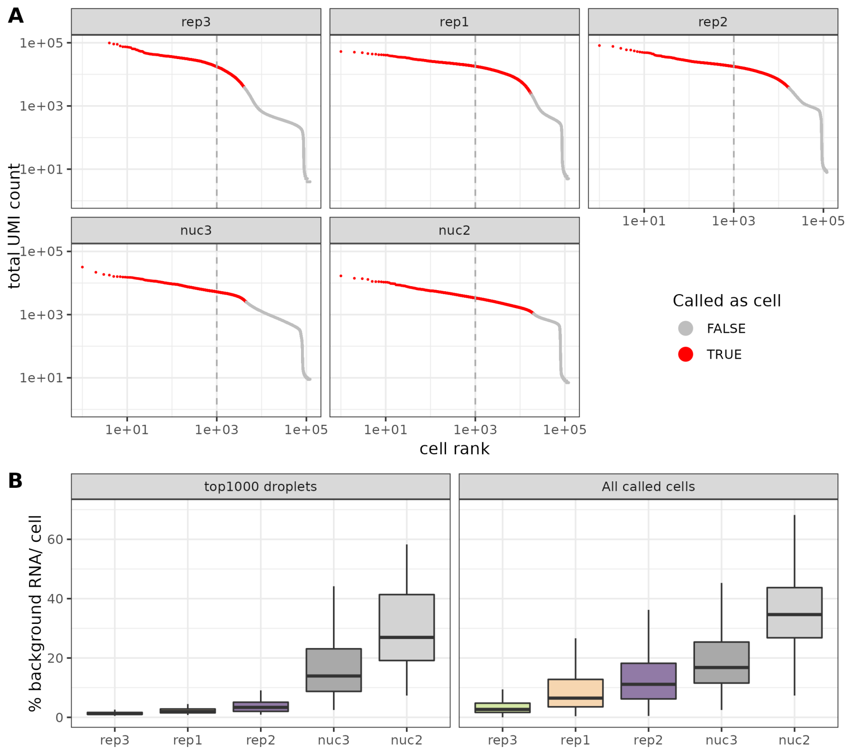

Fig. S4 Definition of true cells and its effect on background noise estimates A) UMI curves showing the total UMI counts for droplet barcodes arranged in descending order. True cells (red) were defined based on a combination of CellRanger cell calls and an inflection point in the UMI curve. The dashed line indicates a hard cutoff of 1000 cells that was used to check the robustness of background noise estimates. B) Estimated background noise levels per cell for the 1000 cells with the highest total UMI counts (left) and for all cells that were defined as true cells based on replicate specific cutoffs (right).

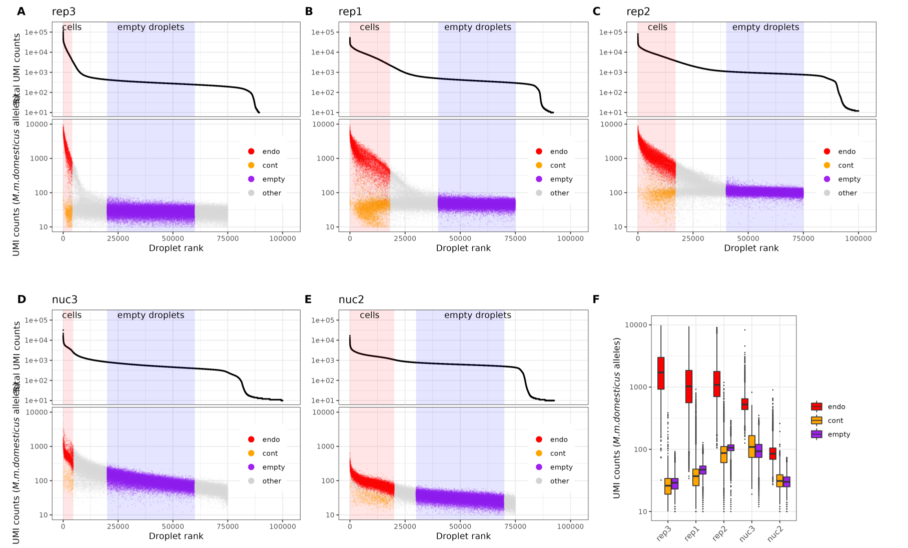

Fig. S5 Definition of endogenous, empty droplet and contamination profiles across replicates. A-E) Droplet barcodes were ordered by their total UMI counts and empty droplets were defined from this UMI curve as barcodes in the low UMI count plateau area (upper panel). UMI counts of reads covering *M. m. domesticus* specific alleles were used to construct three different profiles (lower panel left). *M. m. domesticus* allele counts in *M. m. domesticus* cells were defined as endogenous counts (endo), *M. m. domesticus* allele counts in *M. m. castaneus* cells as contaminating counts (cont) and *M. m. domesticus* allele counts associated with barcodes of the empty droplet plateau as empty droplet counts (empty). F) Boxplots indicate how many UMI counts could be obtained for each profile per droplet barcode.

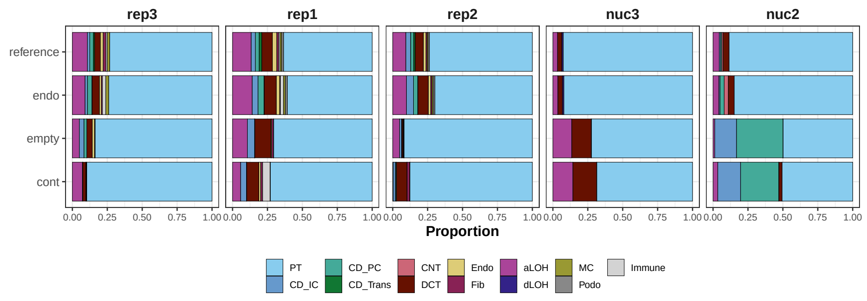

Fig. S6 Dissection of cell type contributions by deconvolution of pseudobulk profiles. The stacked bar plots of 'reference' depict the proportions of cell types in a single cell reference used for deconvolution with SCDC [16]. The 'endo', 'empty' and 'cont' bar plots show the estimated fraction of cell types after deconvolution of pseudobulk profiles that were aggregated for each category.

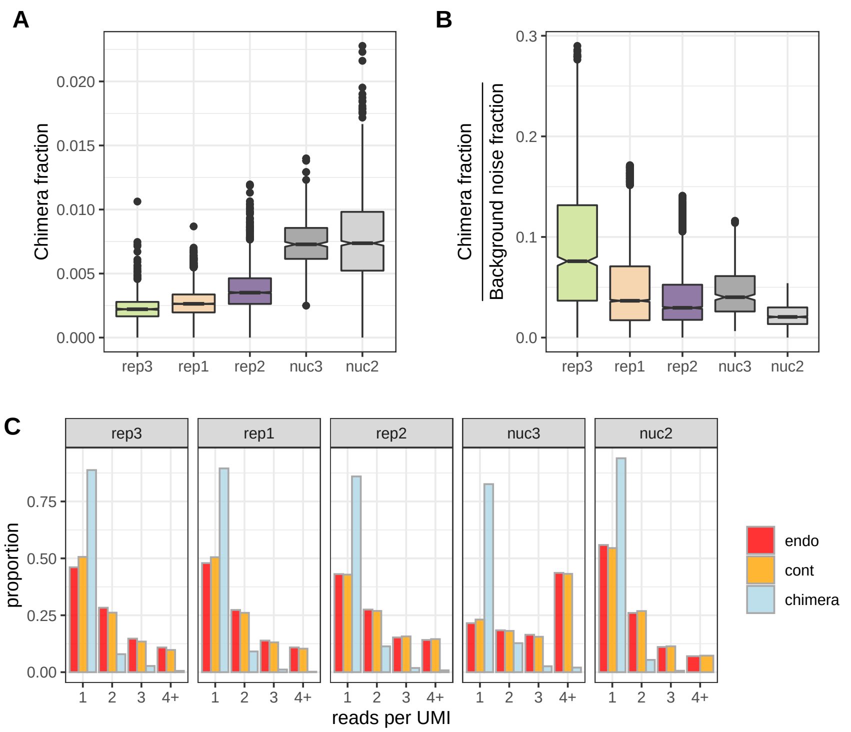

Fig. S7 Identification of barcode swapping due to PCR chimeras. A) Fraction of chimeras per cell. Chimeras were defined as non-unique combinations of cell barcode, UMI and gene. B) Relative fraction of chimeras relative to the estimated level of background noise per cell. C) Distribution of the number of reads per UMI for chimeric molecules, cross-genotype contamination (cont) and endogenous (endo) molecules.

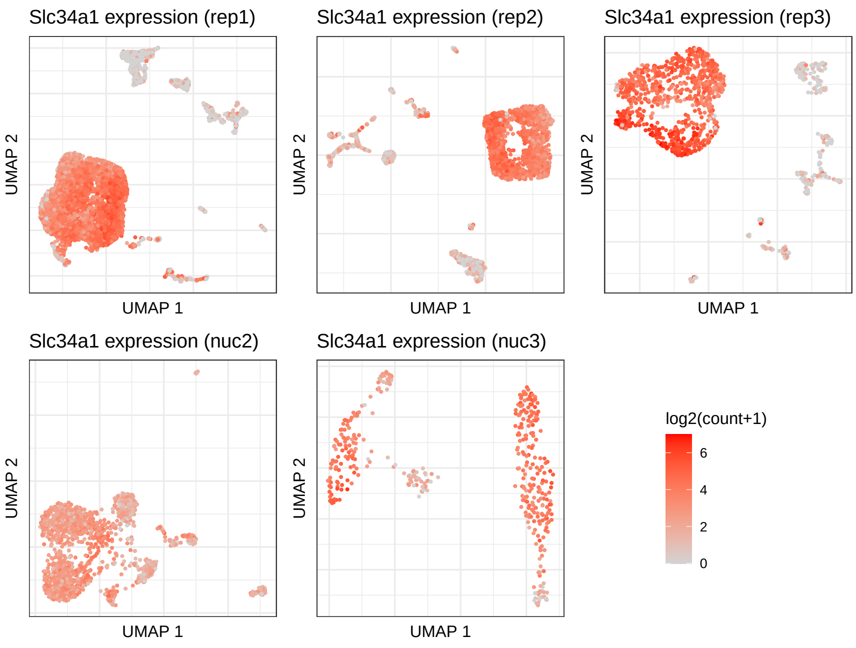

Fig. S8 Slc34a1 expression across replicates. UMAP representation *M. m. castaneus* cells coloured by Slc34a1 expression. Spurious detection of Slc34a1 in all cell clusters is observed in all replicates. In the replicates with the lowest background noise levels (rep1,rep3), Slc34a1 expression is most concentrated in PT cells.

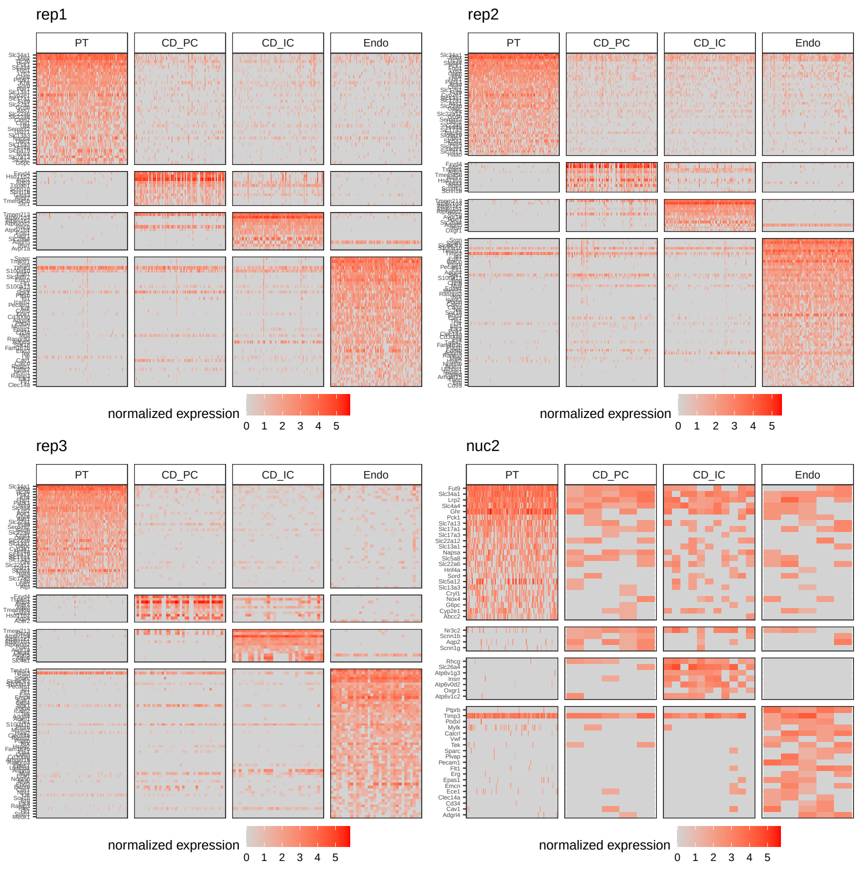

Fig. S9 Expression of cell type marker genes. Heatmaps show the normalized expression of known marker genes for four selected cell types across replicates. Marker genes were obtained from the PanlaoDB database [17] and filtered to select for genes that are detected in at least 50% of the cells of the cell type in which they are expected to be expressed. The replicate nuc3 was excluded from this figure due to an insufficient number of collecting duct and endothelial cells. PT: proximal tubule; CD_IC: intercalated cells of collecting duct; CD_PC: principal cells of collecting duct; Endo: endothelial

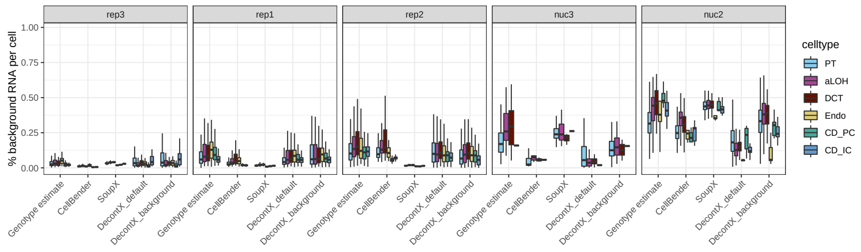

Fig. S10 Estimated background noise levels across cell types. Genotype estimates are inferred based on genetic variants. Cellbender, SoupX and DecontX estimates are calculated for each cell based on a corrected count matrix. PT: proximal tubule; aLOH: ascending loop of Henle; DCT: distal convoluted tubule; Endo: endothelial; CD_PC: principal cells of collecting duct; CD_IC: intercalated cells of collecting duct.

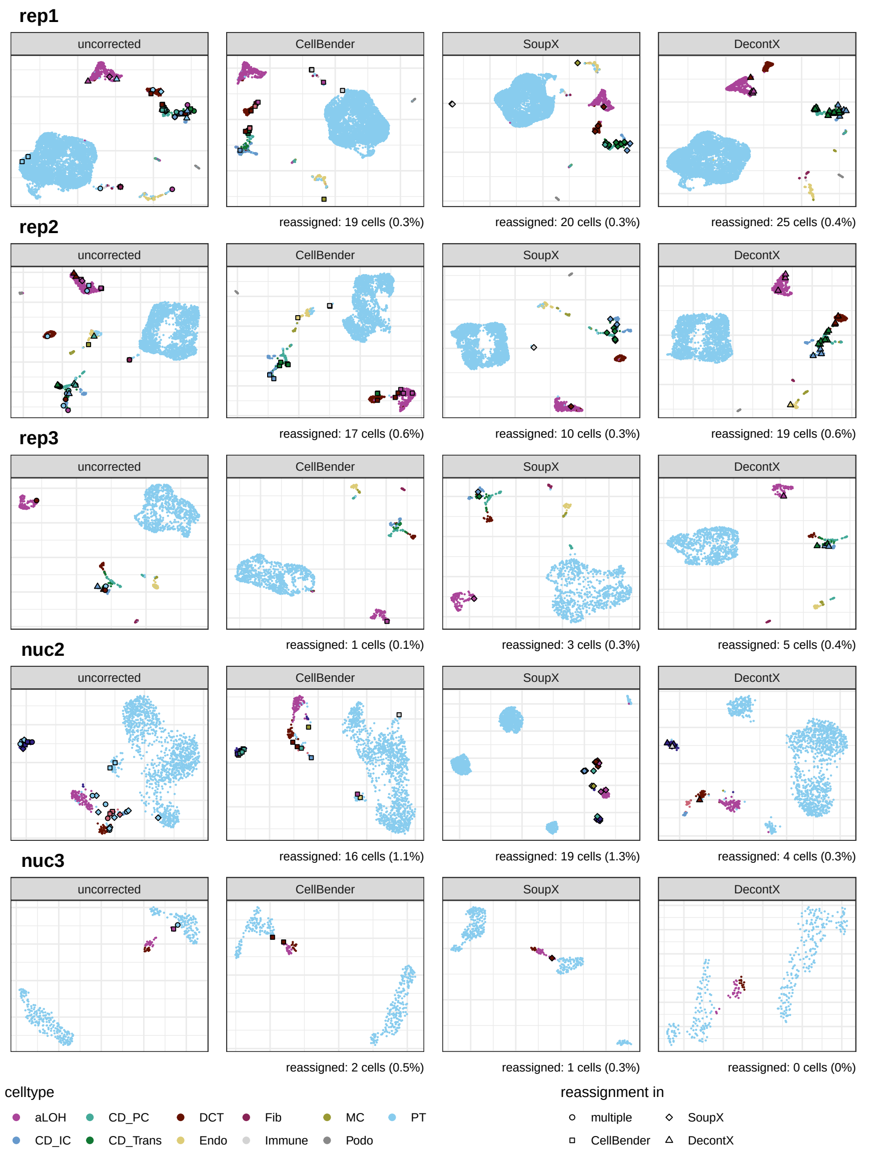

Fig. S11 UMAP representations of all replicates before and after background noise correction. Cells are colored by cell type labels obtained from reference based classification. Individual cells that received a new label after correction are highlighted. In case of the uncorrected data, all cells that received a new label after correction with any of the methods are highlighted. PT: proximal tubule; CD_IC: intercalated cells of collecting duct; CD_PC: principal cells of collecting duct; CD_Trans: transitional cells of collecting duct; CNT: connecting tubule; DCT: distal convoluted tubule; Endo: endothelial; Fib: fibroblasts; aLOH: ascending loop of Henle; dLOH: descending loop of Henle; MC: mesangial cells; Podo: podocytes

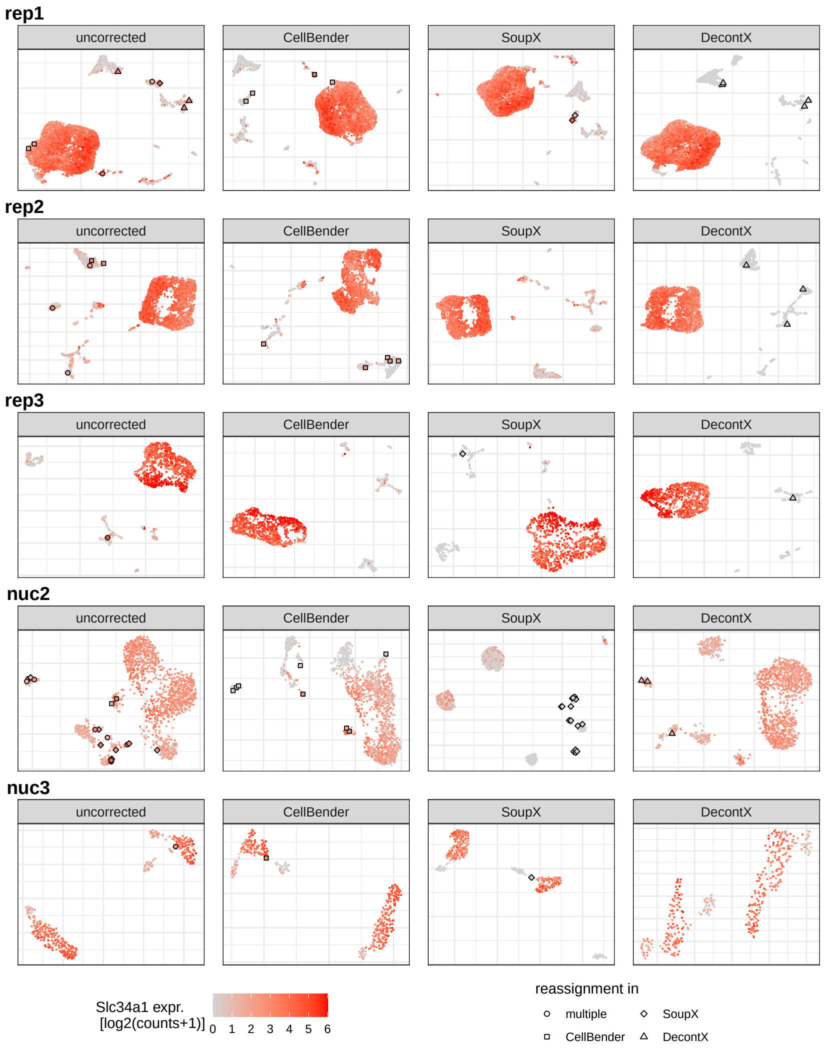

Fig. S12 Detected expression levels of Slc34a1 before and after background noise correction. Cells that were classified as PT cells in the uncorrected data, but got reassigned after correction, are highlighted.

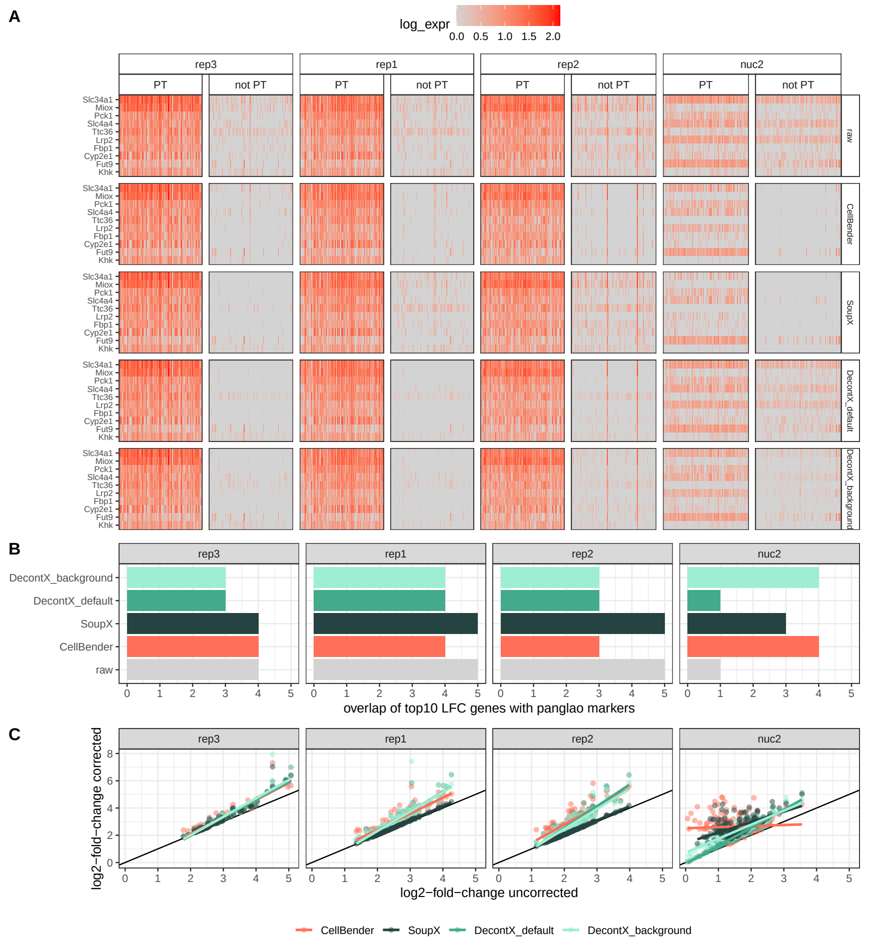

Fig. S13 Effect of background noise correction on marker gene detection. A) Heatmaps depicting the expression of 10 PT marker genes in 100 randomly sampled PT cells and 100 cells from other cell types. The first row of heatmaps is based on the uncorrected count matrix, rows 2-5 on the denoised count matrix output by different methods. B) Overlap of identified and known marker genes. Genes were ranked by log2 fold change between PT an other cells and the overlap of the top 10 genes in this ranking with known marker genes for Proximal Tubule cells from PanglaoDB [17] is shown. C) Log2 fold changes of PangloaDB PT cell marker genes after background noise correction compared to the uncorrected data.

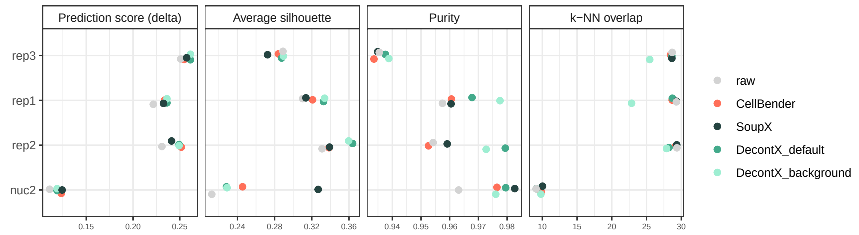

Fig. S14 Evaluation metrics for cell type identification. Prediction score: cell-wise score "delta" of reference based classification with SingleR [21]. Average silhouette: Mean of silhouette widths per cell type. Purity: Cluster purity calculated on cell type lables as ground truth and Louvain clusters as test labels. k-NN overlap: overlap of the k=50 nearest neighbors per cell compared to genotype-cleaned reference k-NN graph.

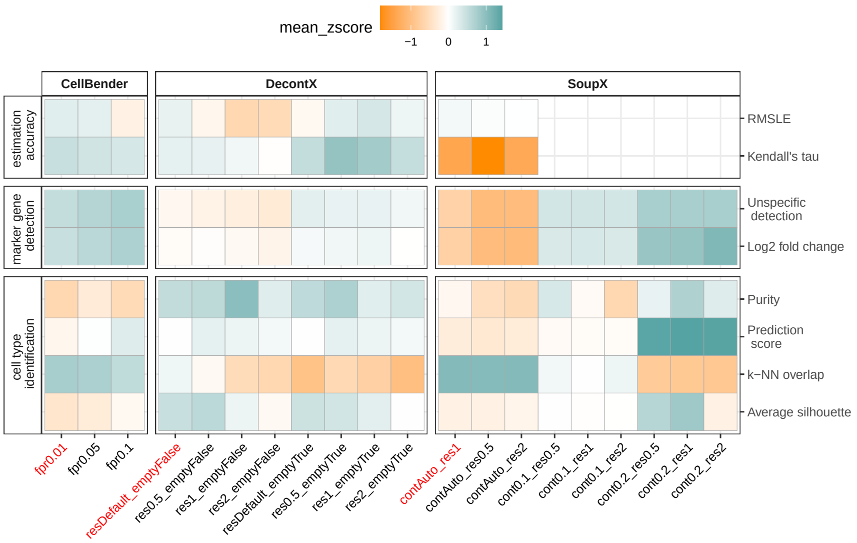

Fig. S15 Evaluation of different parameter settings. Combinations of the most impactful parameter/workflow choices of each method are evaluated. Default parameter settings are highlighted with red font color. For each metric, an average z-score across the replicates rep1, rep2, rep3 and nuc2 is shown, for which higher values indicate better performance. The following parameters were tuned: CellBender: *fpr* (0.01,0.05,0.1); DecontX: cluster lables *z* (resDefault: NULL, res0.5/1/2: vector of cluster labels from Louvain clustering with resolution 0.5/1/2), *background* (emptyFalse: NULL, emptyTrue: provide raw matrix containing empty droplets); SoupX: contamination fraction (contAuto: automatic estimation using *autoEstcont*, cont0.1/0.2: manually set using *setContaminationFraction* (0.1/0.2)), cluster labels (res0.5/1/2: vector of cluster labels from Louvain clustering with resolution 0.5/1/2)
