# Supplementary material for: The effect of background noise and its removal on the analysis of single-cell expression data
Source: Genome Biol. 2023 Jun 19;24:140. doi: 10.1186/s13059-023-02978-x (PMC10278251; doi:10.1186/s13059-023-02978-x)
Supplement: Supplementary file 2 — Additional file 2. Review history. [file 13059_2023_2978_MOESM2_ESM.docx]

Review History

**First round of review**

**Reviewer 1**

**Were you able to assess all statistics in the manuscript, including the appropriateness of statistical tests used?**

Yes, and I have assessed the statistics in my report.

**Comments to author:**

In this manuscript, the authors investigate the effect of 'background noise' (ambient RNA contamination or barcode swapping) in single-cell and single-nucleus RNA-seq data. They generate a realistic benchmarking data set and perform a characterization of the noise as well as an evaluation of tools for its removal.

I found the paper very well written and easy to follow, and I think that the analyses are sensible and provide a balanced and pretty comprehensive investigation of the problem at hand. I also appreciate that the authors provide all the code as well as the processed data on GitHub/Zenodo.

I have only minor suggestions for clarification:

1. In the description of Figure 2, the x-axis is referred to as the 'sequencing depth'. I wonder if a different wording may be more adequate (as the x-axis does not represent the total number of reads sequenced for the cell).

2. I was also wondering whether one might expect differences between cell types in Figure 2 - e.g. since the ability to distinguish reads from the different strains depends on where the informative SNPs are located compared to which genes are in fact expressed in the cell type.

3. I didn't find a description of how the error bars in Figure 3B were calculated (if I understood correctly, there is only one pseudobulk per 'type' - endogenous, contaminating, empty).

4. While the processed data is available on Zenodo, there is no accession number for the raw data (only 'GEO SRPXXXX').

5. Typos and inconsistencies:

a) RNAlater is written in inconsistent ways in different places in the manuscript

b) line 433: could unambigously -> could be unambiguously

c) line 524: Lovain -> Louvain

d) line 582: louvain -> Louvain

e) line 584: used get -> used to get

**Reviewer 2**

**Were you able to assess all statistics in the manuscript, including the appropriateness of statistical tests used?**

No, I do not feel adequately qualified to assess the statistics.

**Comments to author:**

The manuscript by Janssen et al. generated a mixed-subspecies mouse kidney scRNA-seq dataset, and used it to characterize the background noise in 10X Genomics scRNA-seq experiments. The authors demonstrated large variations of background noise between different experiments, and evaluated the potential sources of background noise. Furthermore, the authors benchmarked several algorithms developed to estimate the remove background noise, by evaluating their performance on downstream transcriptome analysis.

Below please find the concerns I have and the suggestions for potential improvements.

1. The quality of nuc2 dataset is concerning in particular, due to low number of genes detected. On the other hand, it is this dataset that has the highest background noise. Besides the variation of 10X Genomics chemistry itself, multiple causes can also lead to this observation, such as a loose cutoff of true cells, suboptimal nucleus preparation, etc. Please provide more detailed explanation for this.

2. It is true that ambient RNA and barcode swapping are the two sources of background noise, but the way authors dissected their effects separately does not totally convince me yet. The authors stated that "the majority of background molecules originates from ambient RNA". This conclusion is majorly based on two observations: 1) "The number of contaminating RNA-molecules depends only weakly on the sequencing depth of the cell"; and 2) "by and large the absolute amount of background noise is approximately constant across cells". However, for 1) statistics is missing. For 2), the authors need to directly compare the absolute contaminated UMI counts in true cells versus in empty droplets. Only if these two values are very close can the authors draw the conclusion.

Here I would also suggest an additional analysis to evaluate potential barcode swapping. Besides, since 10X Genomics provides a 12-bp length (or 10-bp depending on the version) UMI. Except for the extremely highly expressed genes, this UMI space provides adequate complexity even if you pool aggregate the reads of all cells together. For a given relatively lowly expressed gene, the UMI space is enormous. If you observe that the same UMI is captured in more than one cell barcode, it is direct evidence of barcode swapping. The exact barcode swapping can also be estimated. I would strongly recommend evaluating its variation across experiments.

If the new analysis result still points towards that ambient RNA is the major source, the following experimental proof can be done to further strengthen the conclusion. The cells/nuclei can be separate from the ambient RNA-containing supernatant by low-speed centrifugation (such as 300xg), and the RNA content can be quantified using qPCR. The observed background noise is expected close to the sum of barcode swapping (estimated above) and ambient RNA content.

3. For the background noise, the authors did not dissect the effects of sample preparation and the 10X Genomics chemistry itself. It is desired to specify which factor majorly contributes to the large variations of background noise. The two single-nucleus datasets showed very high background noise, 35% and 17% respectively. Noting that intron fraction is lower in empty droplets than endogenous, it is likely ambient RNA leaked from the cytosol, which is likely not efficiently washed away in the authors' protocol. I would like to see whether a series of washing and centrifugation steps will help. And please quantify the ambient RNA content from the supernatant to guarantee that the samples loaded for library prep are qualified.

4. Given that the different principles of background noise estimation algorithms, we might reason that the performance of SoupX DecontX, and CellBender can depend on the noise source of the dataset. The conclusions the authors drew based on rep1-3 are generally applicable, but the conclusions based on nuc2 dataset may not apply to the field in general, unless the authors provide the evidence that their nucleus preparation is reasonably clean.

5. Supplementary Figure S3B is not cited in the text. Its legend is confusing. Please clarify what the panel indicates.

**Authors Response**

**Reviewer #1: In this manuscript, the authors investigate the effect of 'background noise' (ambient RNA contamination or barcode swapping) in single-cell and single-nucleus RNA-seq data. They generate a realistic benchmarking data set and perform a characterization of the noise as well as an evaluation of tools for its removal.**

**I found the paper very well written and easy to follow, and I think that the analyses are sensible and provide a balanced and pretty comprehensive investigation of the problem at hand. I also appreciate that the authors provide all the code as well as the processed data on GitHub/Zenodo.**

**I have only minor suggestions for clarification:**

1. **In the description of Figure 2, the x-axis is referred to as the 'sequencing depth'. I wonder if a different wording may be more adequate (as the x-axis does not represent the total number of reads sequenced for the cell).**

In the text we did refer to the total UMI count covering informative SNPs as a proxy of sequencing depth. However, the reviewer is correct that this might be misleading. We changed the sentence:

“The number of contaminating RNA-molecules (UMIs) depends only weakly on the total UMI counts covering informative variants as a proxy for sequencing depth of the cell.”

2. **I was also wondering whether one might expect differences between cell types in Figure 2 - e.g. since the ability to distinguish reads from the different strains depends on where the informative SNPs are located compared to which genes are in fact expressed in the cell type.**

Thank you for this interesting question. The initial set of genetic variants stems from whole-genome sequencing within the Mouse Genomes Project, hence we do not expect a systematic bias of their location in genes that are expressed across kidney cell types. For the most part, the fraction of UMI counts covering an informative SNP is indeed very similar across cell types (see below and newly added Supplementary Figure S3A). However, we find a slightly higher SNP coverage in proximal tubule cells (PT), which are with 66-89% by far the most frequent cell type. This increase in the SNP detection rate for PTs is probably due to our minimum coverage requirement: Each SNP needs to be covered by at least 20 UMIs. Because there are so many PTs, this threshold can also be reached for genes that are lowly expressed in PTs. While the coverage of informative SNPs per cell type is therefore to some degree dependent on the prevalence of that cell type, this effect is rather small, even for PT cells which are an extreme outlier with respect to cell type frequency. Most SNPs are located in genes that are not very cell type specific.

***Figure S1 Detection of cross-genotype contamination*** *A) Variants that are variable between mouse subspecies were used to identify background noise. Boxplots show the fraction of detected molecules (UMI counts) that contain an informative SNP in each cell across cell types with more than 50 cells. [...]*

3. **I didn't find a description of how the error bars in Figure 3B were calculated (if I understood correctly, there is only one pseudobulk per 'type' - endogenous, contaminating, empty).**

We thank the reviewer for pointing this out. While there is indeed only one pseudobulk per category, the error bars indicate 95% confidence intervals obtained from bootstrapping genes for the calculation of Spearman’s rank correlation. We added this information to the figure legend.

4. **While the processed data is available on Zenodo, there is no accession number for the raw data (only 'GEO SRPXXXX').**

At the time of submission of the manuscript, the screening process from NCBI was not completed yet. The accession number is GSE218853, which is now public and added to the manuscript.

5. **Typos and inconsistencies:**
a) **RNAlater is written in inconsistent ways in different places in the manuscript**
b) **line 433: could unambigously -> could be unambiguously**
c) **line 524: Lovain -> Louvain**
d) **line 582: louvain -> Louvain**
e) **line 584: used get -> used to get**

We thank the reviewer for noticing. The typos are corrected now.

**Reviewer #2: The manuscript by Janssen et al. generated a mixed-subspecies mouse kidney scRNA-seq dataset, and used it to characterize the background noise in 10X Genomics scRNA-seq experiments. The authors demonstrated large variations of background noise between different experiments, and evaluated the potential sources of background noise. Furthermore, the authors benchmarked several algorithms developed to estimate the remove background noise, by evaluating their performance on downstream transcriptome analysis.**

**Below please find the concerns I have and the suggestions for potential improvements.**

1. **The quality of nuc2 dataset is concerning in particular, due to low number of genes detected. On the
   other hand, it is this dataset that has the highest background noise. Besides the variation of 10X Genomics chemistry itself, multiple causes can also lead to this observation, such as a loose cutoff of true cells, suboptimal nucleus preparation, etc. Please provide more detailed explanation for this.**

The reviewer is absolutely right. Issues with cell isolation and nucleus preparation can explain background noise and contribute to the variability between our data sets. Noticeably, we had some difficulties preparing the nuclei suspensions and after observing high noise levels in nuc2, we adjusted the experimental protocol for nuc3 slightly with two additional washing steps.

The 10x Genomics kits for the scRNA-seq and the snRNAseq were all the same chemistry version 3.1.

Generally, we did not mean to comment on the quality of the 10x Genomics method, since we believe that the main source of variation is due to sample properties, such as dissociation conditions for tissue samples, which need to be optimized specifically and are not part of the standard 10x protocol. Hence, we expect such variation in most real life experiments. In order to make this clearer, we rephrased the abstract a little and also added a statement to the discussion (p.9 “Given that every sample ...”). Moreover, for the scope of this study, the inclusion of a low quality sample such as nuc2 is even of value, as it broadens the spectrum of background noise levels and showcases their impact in problematic cases.

On the other hand, we certainly want to avoid variability due to analysis artifacts such as due to too loose cell calling cutoffs. To this end we added an additional Supplementary Figure S4 (see below) to check the impact of a hard-cutoff on cell number, keeping only the 1000 best cells. Even though this stringent cutoff lowered the absolute estimates of background noise, it did not change the ranking of replicates.

***Figure S4 Definition of true cells and its effect on background noise estimates*** *A) UMI curves showing the total UMI counts for droplet barcodes arranged in descending order. True cells (red) were defined based on a combination of CellRanger cell calls and an inflection point in the UMI curve. The dashed line indicates a hard cutoff of 1000 cells that was used to check the robustness of background noise estimates. B) Estimated background noise levels per cell for the 1000 cells with the highest total UMI counts (left) and for all cells that were defined as true cells based on replicate specific cutoffs (right).*

2. **It is true that ambient RNA and barcode swapping are the two sources of background noise, but the
   way authors dissected their effects separately does not totally convince me yet. The authors stated that "the majority of background molecules originates from ambient RNA". This conclusion is majorly based on two observations: 1) "The number of contaminating RNA-molecules depends only weakly on the sequencingdepth of the cell"; and 2) "by and large the absolute amount of background noise is approximately constant across cells". However, for 1) statistics is missing. For 2), the authors need to directly compare the absolute contaminated UMI counts in true cells versus in empty droplets. Only if these two values are very close can the authors draw the conclusion.**

We thank the reviewer for suggesting these analysis and are happy to include them:

Concerning point 1): Correlation coefficients between the endogenous and contaminating UMIs per cell, corresponding to Figure 2B are now provided in Supplementary Table 1. Although these correlations are mostly significant, with Spearman's rho <0.3 they are all relatively weak, especially when compared to the expected correlation for background due to swapping (see new analysis of barcode swapping below).

Furthermore, as Fleming et al. (2019) have argued before, such a weak correlation would even be expected if there was no barcode swapping, simply due to variation in the capture efficiency between droplets. While revisiting these data, we also noticed an inconsistency in the initial filtering steps. The original plots of Figure 2B were based on a less stringent cell filtering. This is corrected now.

Concerning point 2): We now provide boxplots that show that the median number of contaminating UMIs is indeed within the same range as the corresponding allele counts in empty droplets (Figure 3C, Supplementary Figure S5F). Moreover, if the empty droplets have many UMIs in a replicate, there are also more contaminating UMIs. This is consistent with our interpretation that the majority originates from ambient RNA. This is now also mentioned in the results part (p. 5 “The number of contaminating UMI counts ....”).

***Figure S5*** *[...] F) Boxplots indicate how many UMI counts could be obtained for each profile per droplet barcode.*

**Here I would also suggest an additional analysis to evaluate potential barcode swapping. Besides, since 10X Genomics provides a 12-bp length (or 10-bp depending on the version) UMI. Except for the extremely highly expressed genes, this UMI space provides adequate complexity even if you pool aggregate the reads of all cells together. For a given relatively lowly expressed gene, the UMI space is enormous. If you observe that the same UMI is captured in more than one cell barcode, it is direct evidence of barcode swapping. The exact barcode swapping can also be estimated. I would strongly recommend evaluating its variation across experiments.**

We thank the reviewer for this suggestion, however we disagree that the swapping rate can be easily estimated by investigating how often the same UMI is associated with multiple barcodes. For the 10x chemistry that we used here,the 16bp cell barcode is immediately followed by the 12bp UMI, thus most of the time UMI and barcode will be swapped together.

***Response Figure:*** *Construct layout for 10x Single Cell 3’ v3.1(Dual Index) Gene expression Libraries from the 10xgenomics webpage:* [*https://kb.10xgenomics.com/hc/en-us/articles/360000939852-What-is-the-difference-between-Single-Cell-3-and-5-Gene-Expression-libraries-*](https://kb.10xgenomics.com/hc/en-us/articles/360000939852-What-is-the-difference-between-Single-Cell-3-and-5-Gene-Expression-libraries-)

This said we can get an estimate for the amount of barcode swapping by looking for same cell barcode UMI combinations that are associated with different genes as suggested in Dixit et al. (2021). With 0.2-0.7% of chimeric reads (Supplementary Figure S7A) , we find very little evidence for barcode swapping relative to the expected total amounts of background RNA which is 3-35%, the per cell median detected relative swapping fraction relative to the overall background RNA is below 10% for all samples (Supplementary Figure S7B).

Moreover, chimeras are expected to have on average fewer reads per UMI, because they can only start amplification later. Thus if the majority of contaminating reads were to originate from barcode swapping, we would expect that the distribution of reads per UMI for contaminating molecules (cont) is more similar to that observed for chimeras. This is not what we see: The distribution of reads per UMI for contaminating reads resembles the one for endogenous reads much more (Supplementary Figure S7C).

We added a new results section where we describe the chimera detection as evidence for low levels of barcode swapping.

***Figure S7 Identification of barcode swapping due to PCR chimeras.*** *A) Fraction of chimeras per cell. Chimeras were defined as non-unique combinations of cell barcode, UMI and gene. B) Relative fraction of chimeras relative to the estimated level of background noise per cell. C) Distribution of the number of reads per UMI for chimeric molecules, cross-genotype contamination (cont) and endogenous (endo) molecules.*

**If the new analysis result still points towards that ambient RNA is the major source, the following experimental proof can be done to further strengthen the conclusion. The cells/nuclei can be separate from the ambient RNA-containing supernatant by low-speed centrifugation (such as 300xg), and the RNA content can be quantified using qPCR. The observed background noise is expected close to the sum of barcode swapping (estimated above) and ambient RNA content.**

In our experience every additional handling step leads to a radical increase in dead cells or nuclei, hence it is unclear whether the supernatant after a long centrifugation would be representative of the ambient profile levels in a 10x machine, while empty droplets are a direct sample of the supernatant as it exists in the 10x.

Thus sequences obtained from empty droplets provide us with a good way to characterize ambient RNA profiles and amounts. In analogy to the expected results from a qPCR on marker genes, deconvolution analysis provides us with estimates of cell-type frequencies (Figure 3E). Moreover, empty droplets provide us with a whole expression profile for the supernatant, thus allowing a more detailed comparison to the contamination profile, which we report as the correlations. Here, we find that on average the contamination profile is more similar to the profile from empty droplets than the average endogenous one (Figure 3D). In the same vein, we also compared the proportions of intronic reads between empty droplets, endogenous RNA and contaminating RNA and found again that the intronic fraction among the contaminating reads is closer to that of the empty droplets (Figure 3F). Both of those observations lent additional support to the notion that the majority of background RNA originates from ambient RNA.

3. **For the background noise, the authors did not dissect the effects of sample preparation and the 10X Genomics chemistry itself. It is desired to specify which factor majorly contributes to the large variations of background noise. The two single-nucleus datasets showed very high background noise, 35% and 17% respectively. Noting that intron fraction is lower in empty droplets than endogenous, it is likely ambient RNA leaked from the cytosol, which is likely not efficiently washed away in the authors' protocol. I would like to see whether a series of washing and centrifugation steps will help. And please quantify the ambient RNA content from the supernatant to guarantee that the samples loaded for library prep are qualified.**

As we explained for point 1, the goal of this study is not to evaluate protocols, but to characterize the effect that background RNA has on downstream analyses by exploiting the mix of mouse subspecies in a complex data set. For this it is actually advantageous to have variable levels of background noise present in the data. Furthermore, while it is an interesting and independent question how to experimentally reduce noise, we note that our data does represent in our experience a realistic snapshot of snRNA-seq and scRNA-seq data produced from tissues.

Additional washing steps indeed lower the levels of background RNA, we think this is why nuc3 has lower background levels than nuc2 (see Methods). Considering the empty droplet counts as the best proxy for RNA levels of the supernatant, we added boxplots comparing the empty to contamination counts (see above).

4. **Given that the different principles of background noise estimation algorithms, we might reason that the performance of SoupX DecontX, and CellBender can depend on the noise source of the dataset. The conclusions the authors drew based on rep1-3 are generally applicable, but the conclusions based on nuc2 dataset may not apply to the field in general, unless the authors provide the evidence that their nucleus preparation is reasonably clean.**

We rephrased results and discussion, to make it clear that we see the snRNA-seq data as just another possible background RNA profile that can be encountered in a 10x experiment. We hope that this new version now avoids too much generalization. In addition, we removed a mention about the validity of the library preparation methods from the discussion.

Furthermore, we edited the method comparison discussion in a direction that should make it clearer that we are not comparing scRNA-seq and snRNA-seq, but datasets with different relevant features for background removal, which are the cell type composition and how distinct ambient and endogenous profiles are.

5. **Supplementary Figure S3B is not cited in the text. Its legend is confusing. Please clarify what the panel indicates.**

Thank you for pointing this out. We reference it now within the context of which alleles are the most likely source of contamination. Please note that the ordering of Supplementary Figures changed slightly and it is now Supplementary Figure S1B.

**Second round of review**

**Reviewer 2**

The revisions have significantly improved the manuscript. I recommend acceptance for publication in GB.

The newly added analyses have satisfied resolved my concerns, and the descriptions have also rephrased with better clarity. More importantly, the authors settled down that ambient RNA is the major source of background noise, which can be reflected by the reads from empty droplets. The authors further specifically demonstrated the effects of ambient RNA on downstream analysis. These observations provide helpful sample processing and data analysis QC guidance for future investigators.
